# Correlation of precisely fabricated geometric characteristics of DNA-origami nanostructures with their cellular entry in human lens epithelial cells

**DOI:** 10.1186/s11671-025-04188-9

**Published:** 2025-01-22

**Authors:** Yexuan Guo, Tianze Xiong, Hong Yan, Rui Xue Zhang

**Affiliations:** 1 Institute of Medical Research, Northwestern Polytechnical University, 127 West Youyi Road, Xi’an, 710072 Shaanxi China; 2 Xi’an People’s Hospital (Xi’an Fourth Hospital), Shaanxi Eye Hospital, 21 Jiefang Road, Xi’an, 710004 Shaanxi China

**Keywords:** DNA nanotechnology, Ocular delivery, Computer-aided design, Bottom-up, DNA bending rigidity, Mitochondria, Nucleus

## Abstract

Human lens epithelial cells (*h*LECs) are critical for lens transparency, and their aberrant metabolic activity and gene expression can lead to cataract. Intracellular delivery to *h*LECs, especially to sub-cellular organelles (e.g., mitochondrion and nucleus), is a key step in engineering cells for cell- and gene- based therapies. Despite a broad variety of nano- and microparticles can enter cells, their spatial characteristics relevant to cellular uptake and localization remains elusive. To investigate cellular internalization of nanostructures in *h*LECs, herein, DNA nanotechnology was exploited to precisely fabricate four distinct, mass-controlled DNA-origami nanostructures (DONs) through computer-aided design. Ensembled DONs included the rods, ring, triangle, and octahedron with defined geometric parameters of accessible surface area, effective volume, compactness, aspect ratio, size and vertex number. Atomic force microscopy and agarose gel electrophoresis showed that four DONs self-assembled within 3.5h with up to 59% yield and exhibited structural intactness in cell culture medium for 4 h. Flow cytometry analysis of four Cy5-labelled DONs in *h*LECs HLE-B3 found time-dependent cellular uptake over 2 h, among which the octahedron and triangle had higher cellular accumulation than the rod and ring. More importantly, the vertex number among other geometric parameters was positively correlated with cellular entry. Confocal images further revealed that four DONs had preferential localization at mitochondria to nucleus at 2 h in HLE-B3 cells, and the degree of their biodistribution varied among DONs as evidenced by Manders’ correlation coefficient. This study demonstrates the DONs dependent cellular uptake and intracellular compartment localization in *h*LECs, heralding the future design of structure-modulating delivery of nanomedicine for ocular therapy.

**Graphical abstract:**

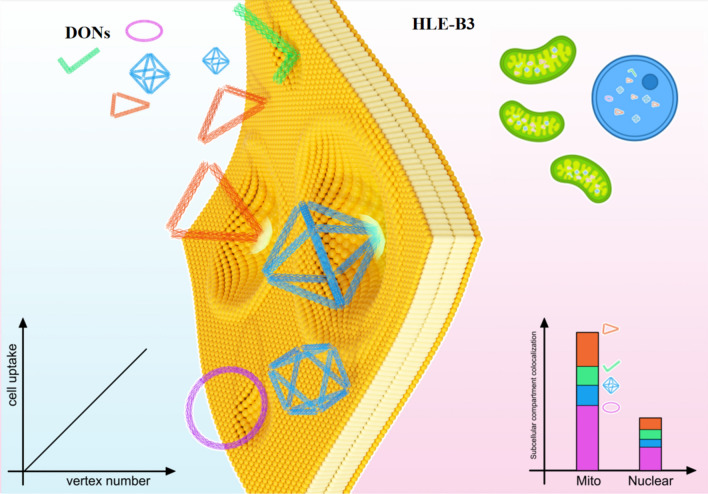

**Supplementary Information:**

The online version contains supplementary material available at 10.1186/s11671-025-04188-9.

## Introduction

Human lens epithelial cells (hLECs) is critical for the transparency of ocular lens, and aberrant gene expression in hLECs can lead to opacity in the crystalline lens, known as cataract [1–3]. Posterior capsule opacification (PCO) (called secondary cataract), for example, is a common complication after cataract surgery, and its development involves fibrotic processes of residual hLECs in response to the surgical wound in the anterior capsule [4]. Under oxidative stress, several molecular signaling pathways associated with hyperproliferation and epithelial-mesenchymal transition of hLECs are activated, including TGF-β, Wnt/β-catenin and ERK1/2, eventually leading to PCO formation [4–6]. Typically, these pathological processes occur at nucleus and mitochondria [6, 7]. Although chemical compounds, such as antioxidants or sterols (e.g., lanosterol), have been shown to reverse cataract by scavenging free radicals or dissolving crystallin aggregates in ocular lens, respectively, there is no approved pharmacotherapy available for cataract and PCO [8, 9]. Currently, an eyedrop containing antioxidant N-acetylcarnosine (Can-C™) has shown promising improvement in control of senile cataract in clinical trial [10]. In addition, various ocular nanomedicine and devices have been studied for their ability of reaching the intraocular space by overcoming specific ocular barriers (e.g., tear film, blood-aqueous) in the anterior segment [11–14]. However, formulation therapeutics that directly reach ocular cells (e.g., hLECs) and even target intracellular compartments (e.g., mitochondria and nucleus) is limited. Our previous study prepared a series of spherical polymeric poly lactic-co-glycolic acid (PLGA) nanoparticles, and found that varied drug release rates had the impact on cellular uptake in hLECs activity, but cellular entry process of PLGA nanoparticles cannot be elucidated due to different sizes, varied polymer compositions and degradation rate [15].

Intracellular delivery of target materials (e.g., nucleic acids and peptides) using the particles, especially at the level of sub-cellular organelles (e.g., mitochondria and nucleus), is a key approach to engineering diseased cells (e.g., *h*LECs and cancer cells) for cell- and gene- based therapies [16–18].The number of particle parameters, such as size, shape, modulus, surface charge and chemistry, play an important role on cellular entry and trafficking [19–21]. However, owing to the limited technique of producing non-spherical particles, the impact of the key particles’ spatial variables, such as the surface area and volume, on cellular entry remains elusive. So far, the “top-down fabrication” is the main approach to prepare non-spherical particles, including ab initio synthesis (e.g., lithography and microfluidics) and tailoring of original spherical particles [22]. For example, cellular internalization via phagocytosis in the alveolar macrophages was critically affected by target geometry of polystyrene microparticles (0.5–3 µm) prepared from spherical particles using the film-stretching technique [23, 24]. Gratton et al. used a particle-replication in non-wetting template (PRINT) method to synthesize particles varying in both size (100 nm to 5 µm) and shape (i.e., cube and cylinder) at a constant surface charge and mass, and found that non-spherical particles used different mechanisms of endocytosis to enter HeLa cells and the high aspect-ratio (AR) particles had faster internalization than ones with more symmetrical low AR [25]. Yet, fabrication of those multicomponent polymeric particles yielded the size greater than 100 nm, making difficult gain access to the intended intracellular compartment, such as nucleus.

DNA-origami nanostructures (DONs) has become a facile tool to explore the non-spherical particle design with near-atomic resolution for cell internalization and trafficking. The DNA-origami method was firstly reported by Rothemund in 2006, in which DONs rely on folding a long single stranded DNA (*ss*DNA) (scaffold) with hundreds of designed short *ss*DNA (staples) via Watson–Crick base pairing [26, 27]. The principle of DONs was based on Seeman’s theory of using the holliday junction to generate three-dimensional (3D) lattices of nucleic acid [28]. Compared to multicomponent self-assembly of polymers, unimolecular folding of *ss*DNA by “bottom-up fabrication” imparted well-defined mass and geometric dimensions to DONs at the nanoscale [29]. Bastings et al. examined the cellular uptake of 11 customized DONs (i.e., rod, ring and block, barrel and octahedron) and found that their shape and AR significantly influenced the cellular uptake across different cell types [30]. Cell trafficking has also been studied using DONs. For example, transmission electron microscopic images of a gold nanoparticle tagged rod-shape DONs revealed four-stages of endocytosis process in cancer cells, where the DONs first rotated to align the membrane longitudinally for scavenger receptors binding and then entered the cell in a perpendicular orientation [31]. A recent study reported the nucleus delivery of a 30 nm DONs nanorod by conjugating anti-RNA-Polymerase II antibodies against nuclear factors, and demonstrated that DONs remained intracellular intact for 24 h and exhibited sub-diffusive motions in the nucleus of human osteosarcoma cells, suggesting an effective strategy of reaching the nucleus [32].

**Fig. 1:**
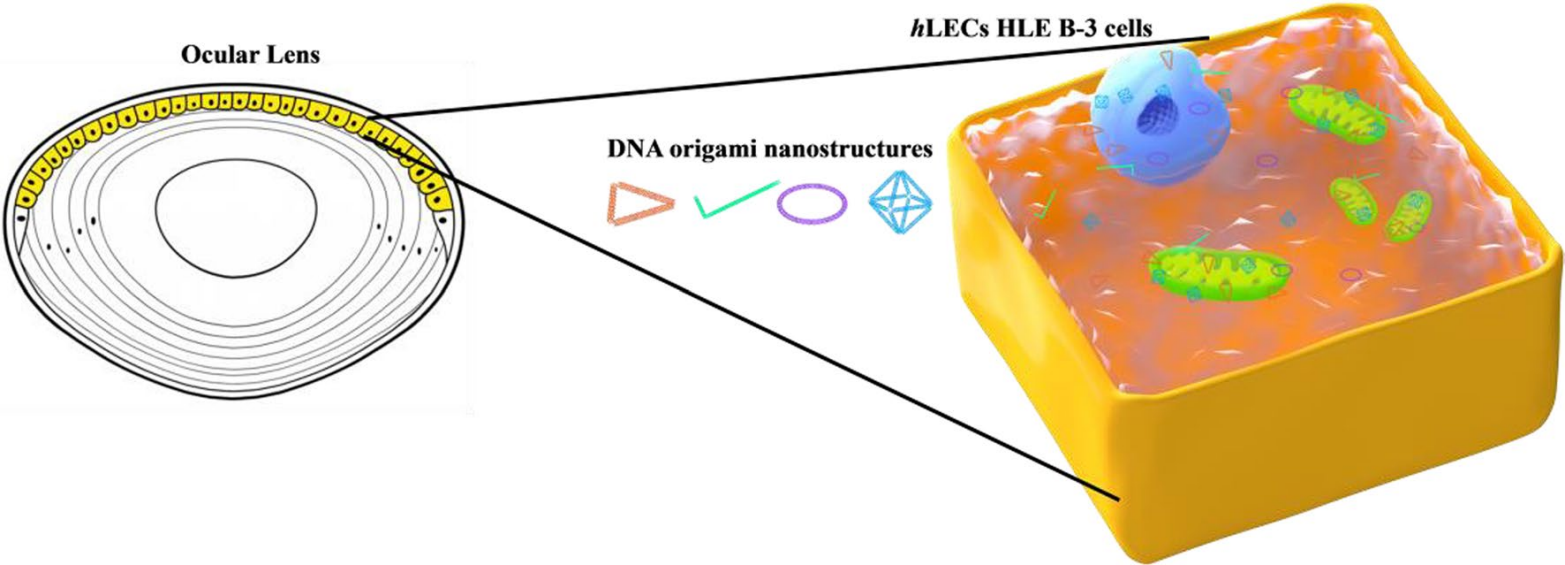
Uptake of DNA origami nanostructures in human lens epithelial cells (*h*LECs). Left panel: *h*LECs form a single layer of cuboidal epithelial cells (yellow colored) that are located beneath the anterior capsule and extend behind the equator, and they are mainly responsible for regulating the physiological homeostasis of the lens, thus maintaining the function and structure of the lens. Right panel: Four classic DONs geometries (triangle, rod, ring and octahedron) and their uptake in hLECs HLE B-3. Four DONs are colored with orange for triangles, green for rods, purple for rings and blue for octahedrons, respectively

To investigate the cellular entry capacity of the particle in *h*LECs, in the present work, DNA-origami technology was applied to fabricate four DONs with distinct six geometric characteristics, including the accessible surface area (ASA), effective volume (EV), compactness, AR, size and vertex number (Fig. 1). Dimensions of the 1D-rod, 2D-ring and triangle and 3D-octahedron were prepared through the computer-aided design (CAD) followed by annealing reaction of scaffold and staples ssDNA strands at programmable temperature. Agarose gel electrophoresis (AGE) and atomic force microscopy (AFM) were used to characterize the yield, purity, intactness and structure of four DONs. To evaluate cell uptake and localization in *h*LECs HLE-B3 cells, each DONs was incorporated and calibrated with three fluorophore Cyanine5 (Cy5) labelled staples. The flow cytometry and confocal laser scanning microscopy (CLSM) were used to determine intracellular accumulation and biodistribution of four DONs. Finally, the Pearson’s correlation and Mander’s correlation coefficient (MCC) were applied to analyze the geometric and structural correlation of DONs with cell uptake and co-localization at sub-cellular organelles, mitochondria and nucleus.

## Materials and methods

### Reagents and supplies

Agarose and Gel Stain were purchased from TransGen Biotech (Beijing, China). Poly-l-Lysine (PLL) was purchased from Novonature (Shaanxi, China). Single-stranded DNA (ssDNA) scaffold M13mp18 (7249 nucleotides (n.t.), 100 nM) was purchased from New England Biolabs, China and kept frozen at − 20 °C as small aliquots. ssDNA staples (11 n.t. ~ 43 n.t., 100 μM) and fluorophore Cy5-labelled DNA staples (100 μM, 3′ was modified by covalent linking) purified by High Affinity Purification (HAP) were purchased from Sangon Biotech (Shanghai, China) and stored at − 20 °C. DNA Ladder (DL 5000, 100 base pairs (bp)) was purchased from Vazymz (Nanjin, China). All DNA sequences were provided in Supplementary Material (Suppl. Mater.) S1. Magnesium Chloride Hexahydrate (MgCl_2_·6H_2_O) was purchased from Solarbio Life Sciences (Beijing, China). Ultrapure water was obtained from Milli-Q Synergy with UV Water Purification System, USA. Sterile phosphate buffered saline (PBS) (1×) was purchased from HAT (Shaanxi, China). MitoTracker™ Green FM, 4′,6-diamidino-2-phenylindole (DAPI) and ultrapure 10 × Tris–Acetate EDTA (TAE) Buffer (400 mM Tris Acetate and 10 mM EDTA) were purchased from Thermo Fisher Scientific (Massachusetts Waltham, USA). Fetal bovine serum (FBS) was purchased from Shuangru Biotech (Shanghai, China). Minimum essential medium Eagles with Earle’s Balanced Salts (MEM/EBSS) and penicillin–streptomycin solution (100×) were bought from Hyclone, USA. Eight-Tube PCR strips (200 μL) were bought from KIRGEN (Shanghai, China). Six-well plate culture dishes (Polystyrene, 35 mm, 9.5 cm^2^) and confocal imaging petri-dishes (Polystyrene, 35 mm, 0.17 mm glass-bottom) were purchased from NEST Biotechnology Co., Ltd (Wuxi, China).

### DONs design and simulation

The design process of four DONs (i.e., rod, ring, triangle and octahedron) used two open-source software packages based on the description by Douglas et al. and Suma et al. [33, 34]. First, the caDNAno (version 2.4.0, Wyss Institute, Harvard University) was used for prototyping of DNA nanostructures constrained to a honeycomb framework. In this step, a continuous raster-style scaffold path of the target structure was populated with the M13mp18 ssDNA vector (7249 n.t.), and the complementary staple sequences with 10 to 50 bases long were determined. Then, the TacoxDNA (webserver: http://tacoxdna.sissa.it/cadnano_oxDNA, Institute for Computational Molecular Science, Temple University) was applied to covert the caDNAno generated structure in *json* file format to an oxDNA configuration followed by running short relaxation protocol for molecular dynamics (MD) simulations of four DONs in spatial scale. A sequence-average model in oxDNA2 was used to model the general properties of each DONs, such as the basic interactions at a nucleotide-level model, and the overall global structure of the accurately determined DONs within the experimental resolution. A MD simulation for individual DONs involved the relaxation of initial geometry by first running a minimization algorithm for a few thousand steps followed by a relaxation algorithm requiring a larger-scale motions of atoms in an origami shape. A MD simulation using a modified backbone potential (max_backbone_force = 5) was used to allow larger-scale motions. The results of oxDNA simulation were visualized on a freely available webserver oxdna.org (webserver: https://oxdna.org/static/oxdna-viewer/index.html, oxDNA 2.0, University of Oxford) [35]. Suppl. mater. S2 provided four DONs design of their DNA scaffold routes.

### Synthesis and purification of DONs

DONs were created via self-assembly in one-pot containing the folding buffer according to Rothemund, Dey et al. and Beshay et al. with modification [26, 27, 36]. Folding buffer recipes of each DONs (rod, ring, triangle and octahedron) were provided as Suppl. Mater. S3. In brief, the final folding buffer of all four DNA nanostructures was prepared in the 8-Tube PCR strip containing the mixture of 1.6 nM of linear scaffold ssDNA M13mp18, 160 nM of short staples (up to 234 strands) and 12.5 mM MgCl_2_ in a 100 µl volume of 1 × TAE buffer (diluted with ultrapure water). The annealing reaction was performed in a thermal cycler (C1000 Touch, Bio-Rad, China) with the desired programable temperature set-up as the following: 95 °C for 5 min and ramp from 95 to 20 °C at a rate of 0.5 °C/min. The total annealing time was 3.5 h. The assembled DNA nanostructure samples were stored at 4 °C in a refrigerator. Excess staples were removed by ultracentrifugation, in which the ultrafiltration tube (500 µl, 100 kDa, regenerated cellulose membrane) (Merck KGaA, German) was spun at 5000 × g for 8 min at 4 °C. The membrane was then rinsed by adding 1 × TAE with 12.5 mM MgCl_2_ buffer, diluting the sample to 500 µl and repeating the previous centrifugation parameters. The process was repeated twice. Finally, the sample was recovered at the bottom through inverting the inner membrane device and then spinning it at 10,000×*g* for 2 min. The purified DNA-origami sample was diluted to 1.6 nM in 50 µL 1 × TAE buffer containing 12.5 mM MgCl_2_. Cy5-labelled DONs were prepared in the same way except that three covalently attached Cy5-fluorophores DNA staples (the sequences were provided in Suppl. Mater. S6) were incorporated in the DONs.

### Ensemble characterization by nanodrop and AGE

To assess self-assembly performance, purified DONs were quantified using NanoDrop One microvolume UV–vis spectrophotometer (Thermo Fisher Scientific Inc, USA) and AGE, respectively. To estimate the yield percent (%) of four DONs, one µL of a purified DONs sample was dropped on the pedestal of NanoDrop instrument, and DNA concentrations were measured at UV absorbance 260 nm under the double-stranded DNA detection module. Ultrapure water was used as the blank. Yield% of DONs was calculated according to the Eq. (1) (Suppl. Mater. S4):

1 $$DONs\,\,Yield{\text{\% }} = \frac{Measured\,\,concentration\,\,of\,\,DONs}{{Theoretical\,\,value}} \times 100{\text{\% }}$$

The integrity and purification of DONs were analyzed by AGE using Thermo Scientific EasyCast Mini Gel System apparatus. In brief, six μL of each DONs or scaffold strand was mixed with 1 μL of loading buffer and then loaded into the 1.2% agarose gel (running buffer: 1 × TBE, 12.5 mM MgCl_2_). The samples were allowed to migrate at 100 V for 45–60 min at 25 °C. GelDoc XR + Gel documentation system and Image Lab 6.0 (Bio-Rad China) was used for the gel band visualization and captured AGE images results were analyzed by Image J2 (https://imagej.net/ij, USA). The purification efficiency of DONs was determined according to the Eq. (2) (Suppl. Mater. S5):

2 $$DONs\,Purity{\text{\% }} = \frac{OD\,of\,DONs}{{OD\,of\,sum{ }\left[ {DONs\, + \,staple{ }\,\,DNA} \right]{ }}} \times 100{\text{\% }}.$$

### AFM structure characterization

Purified DNA-origami samples (1.6 nM) were diluted 4 × with 1 × TAE containing 12.5 mM MgCl_2_. Then, 20 µl of samples was deposited onto a freshly cleaved PLL-coated mica surface (ongjingkeyi Technology Co., Ltd, Beijing, China). To coat with positively-charged PLL, the mica was incubated with PLL (0.1 g/ml) for 30 min at room temperature, and nitrogen-blow dried at platform of Nitrogen Generator (PGN-16L, PULAIXI, China) according to a previous study [37]. After incubation for 15 min, the sample deposited PLL-mica was rinsed with ultrapure water to remove any unbound DNA and further dried under a gentle stream of nitrogen. The DNA nanostructures were obtained at room temperature using atomic force microscopy (AFM) (Dimension Icon, BRUKER, German) equipped with a silicon tip on a nitride cantilever (SNL-10, tip radius 10 nm) (ScanAsyst-Air, German). Imaging model of AFM was ScanAsyst-Air. Samples were scanned with a scanning angle of 0°, scanning speed of 1.0 Hz, Peak Force setpoint 0.024 V, Peak Force Amplitude 150 nm, Peak Force Frequency of 2 kHz and the Spring Constant of 0.4N/m.

### Stability and Cy5-fluorescence calibration of DONs

To prepare DONs compatible for cell uptake, freshly prepared culture media (MEM/EBSS supplemented with 20% FBS) was mixed with DONs samples at 1:1 (v/v) (e.g., 5 μL) in the 8-Tube PCR strip. The tube strip was placed in the thermal cycler at 37 °C, and at pre-determined time points of 0 min, 1 h and 4 h, the samples were taken out of the thermal cycler for AGE analysis to determine the global intactness of DONs.

To enable comparison of the fluorescent data among four DONs, the fluorescence calibration for each Cy5-labelled DONs (rod, ring, triangle and octahedron) was performed [38, 39]. Firstly, the purified DONs concentration was measured by Nanodrop. Then, to experimentally determine the number of Cy5 labelled DNA oligos (sequences of Cy5-oligos referred to Suppl. Mater. 6) incorporated into individual DONs, fluorescent intensity of 10 µL of DONs samples in triplicate was added into a black 384-well plate and measured by a multi-functional enzyme-linked immunosorbent assay analyzer (SPARK, Tecan, Switzerland) at excitation 630 nm and emission 670 nm (the standard curve of Cy5 fluorescence referred to Suppl. Mater. 6.). The fluorescent intensity of DONs triangle was chosen as an internal standard to calculate the correction factor for other nanostructures (rod, ring and octahedron). The correction factor was calculated according to the equation (Suppl. Mater. S7):

$$Correction\,\,factor = \frac{{{\text{Fluorescence}}\,\,{\text{intensity}}\,\,{\text{of}}\,\,{\text{DONs}}}}{{{\text{Fluorescence}}\,\,{\text{intensity}}\,\,{\text{of}}\,\,DONs_{triangle} { }}} \times \frac{{{\text{Molar}}\,\,{\text{concentration}}\,\,{\text{of}}\,\,{\text{DONs}}}}{{{\text{ Molar}}\,\,{\text{concentration}}\,\,{\text{of}}\,\,DONs_{triangle} { }}}$$

Dynamic light scattering (DLS) was further used to measure the particle size distribution of Cy5-labelled DONs before and after purification and at while incubating in the MEM supplemented with 10% FBS at 37 °C for 4 h. 100ul of tenfold concentrated DONs samples (0.002 mg/ml) were filtered using a syringe attached with a polyethersulfone membrane filter with pore size of 0.22 μm and diameter of 8 mm. The hydrodynamic size of DONs samples was measured by the NanoBrook 90Plus PALS instrument (Brookhaven Instrument, USA).

### Cell culture of hLECs HLE B-3

The human lens epithelial cell line HLE-B3 (passage 21, BNCC, China) were cultured with a continuous layer of cells (monolayer layer) in the MEM supplemented with 10% FBS, 2 mmol/L L-glutamine, 1% penicillin and streptomycin in a 55 cm^2^ Petri-dish (Corning, USA) at 37 °C in a humidified incubator supplied with 5% CO_2_ and 100% relative humidity (Forma 371, ThermoFisher, USA). When cells reached 80–90% confluence, they were trypsinized and sub-cultured at threefold dilution.

### Flow cytometry measurement of cellular uptake

HLE-B3 cells were seeded on the 6-well plate culture dishes (0.5 × 10^6^ cells per well) and allowed to grow for 12 h. Before any treatment, cells were starved in serum-free medium for additional 2 h. Cells were then incubated with individual Cy5 labelled DNA nanostructure at a concentration of 0.4 nM (diluted in 1 × TAE and 12.5 mM MgCl_2_). At pre-determined time points of 5 min, 15 min, 30 min, 45 min, 60 h, and 120 h, cells were washed with PBS to stop further uptake. Cells were then trypsinized and collected in Eppendorf tubes with PBS and stored at 4 °C. Note that cell sampling procedure were conducted in a consistent manner. Cells prepared for flow cytometry were filtered through a cell strainer (nylon mesh) to eliminate clumps and debris and were analyzed directly on flow cytometer (BD FACSAria™, USA). Cy5 fluorescence was detected by tuning the laser at excitation 640 nm at 40 mW, and emission was detected using the filter bandpass (670 nm at 30 mW). Untreated HLEB-3 cells were used as the control. Cells of 3 × 10^4^ were acquired per sample in triplicates. FlowJo_v10.8.1 software (Tree Star, Ashland, OR) was used to gate non-damaged cell population and obtain the Cy5 fluorescence intensity for DONs uptake in HLE-B3 cells.

### CLSM for intracellular biodistribution

HLE-B3 cells were seeded on the confocal imaging peri-dish (0.1 × 10^6^ cells per well) and allowed to grow for 12 h followed by starvation in the serum-free medium for additional 2 h. Cells were then incubated with various Cy5-labelled DNA nanostructure at the concentration of 0.4 nM (diluted in 1 × TAE and 12.5 mM MgCl_2_) for 2 h at 37 °C. The Cy5-labelled ssDNA staples (40–42 n.t. and 1.2 nM was used a control. HLE-B3 cells were washed three times with PBS, and mitochondria and nuclei were labeled with the MitoTracker™ Green FM (200 nM) for 30 min and DAPI (20 μM) for 15 min at 37 °C, respectively. The stain solution was removed and cells were rinsed three times in pre-warmed 37 °C PBS ready for CLSM imaging. Cell distribution of DNA nanostructures were imaged using a confocal laser scanning microscope (CLSM) (FV3000, Olympus, Japan, resolution: 120 nm) in the following microscopic settings: excitation wavelength of 405 nm(blue channel), 488 nm (green channel) and 648 nm (red channel) laser to visualize nuclei (blue), mitochondria (green) and Cy5-labelled DONs (red), respectively; Objective Lens of UPLSAPO 60XO, Objective Lens Magnification of 60.0×  and Objective Lens NA of 1.35. All CLSM images were collected by FV31S-SW (Olympus, Japan), with signal threshold settings of a minimum of 100 and a maximum of 4000.

### Pearson correlation between geometry and cellular uptake

A one-tailed *Pearson* correlation was performed to analyze the correlation between cell uptake capacity and different geometric parameters, including vertex number, Size, Effective volume, Compactness, Aspect ratio, and Accessible surface area. Both cellular uptake percent (%) and the total fluorescence intensity obtained from the flow cytometry analysis at pre-determined time points of 5, 15, 30, 45, 60, and 120 min was used to correlate with geometric parameters of each DONs. The correlation coefficient *R* value and *P* value were used to measure the linear correlation between two variables.

### MCC analysis of co-localization in confocal images

The MCC method was used to quantitatively analyze the degree of colocalization of DNA nanostructures at mitochondria and nucleus according to Manders et al. [40]. In brief, after remove the background and offset, Coloc 2 Fiji’s plugin in ImageJ was employed to perform the pixel intensity correlation. For each of DNA nanostructures (rod, ring, triangle and octahedron), 6 confocal images for individual DNA nanostructures were used, and we selected 67, 68, 62 and 117 cells using the region of interest (ROI) manager. Co-localization coefficients (M_1_ and M_2_) were obtained: M_1_ described the proportion of mitochondria or nucleus that was colocalized with each DONs, while M_2_ described the proportion of each DONs that were colocalized with mitochondria or nucleus. Both values of M_1_ and M_2_ closer to 1 indicated the high degree of co-localization relation between Cy5 fluorophore for DONs and DAPI and Mitotracker Green FM for nucleus and mitochondria staining, respectively.

### Statistical analysis

All data were analyzed using GraphPad Prism (version 9.02, USA) or Excel (Microsoft, Redmond, VA) and presented as mean ± standard deviation (SD). All images were drawn using GraphPad Prism. Ordinary one-way ANOVA was used to compare means between groups. The statistically significant differences were defined as *p < 0.05, **p < 0.01, ***p < 0.001 and ****p < 0.0001.

## Results and discussion

### Geometric design of four DONs

**Fig. 2:**
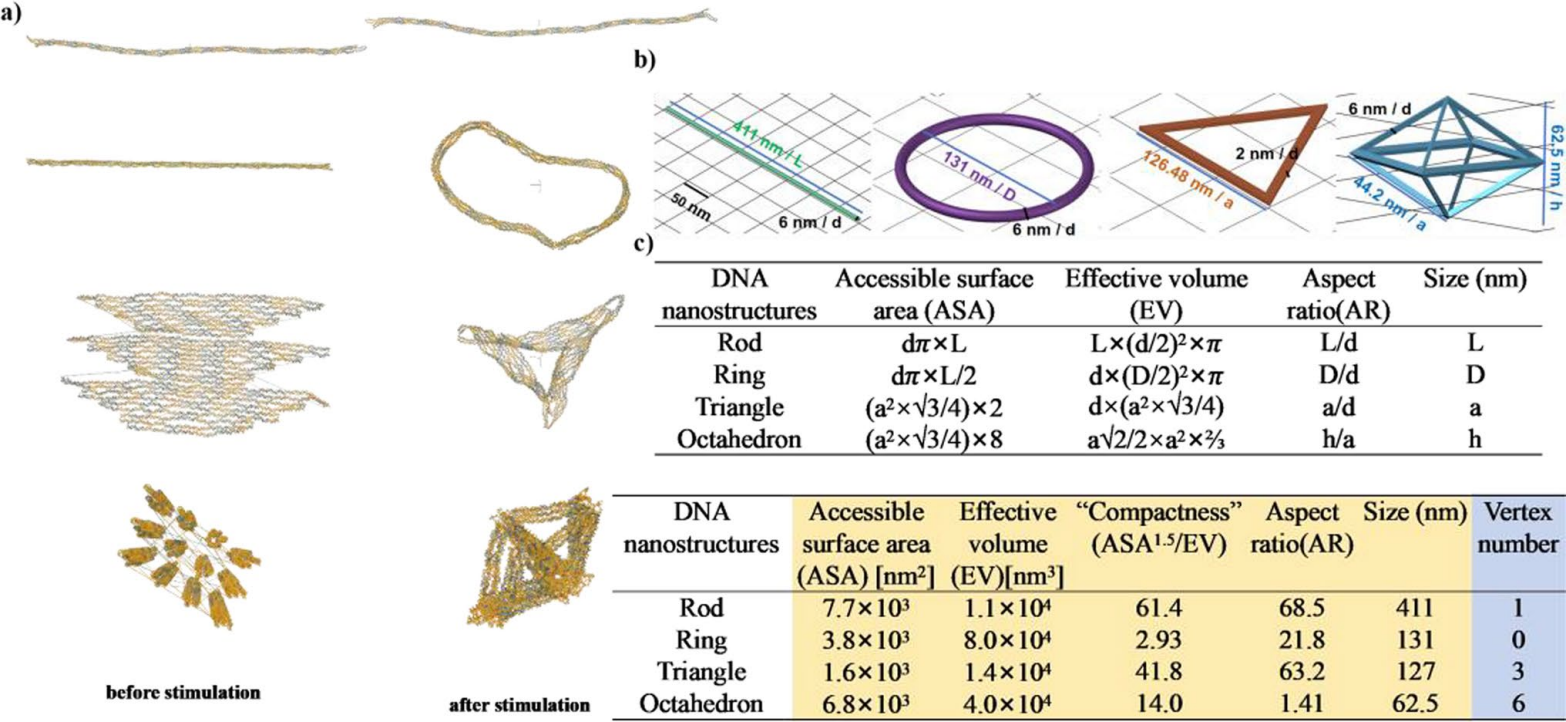
Visualization of designed four DONs and calculation of their geometric parameters. **a** oxDNA2.0 MD simulations of the nanorod (1D), ring (2D), triangle (2D), and octahedron (3D). A graphical web server (oxDNA.org) was used to spatially visualize DNA structures before and after simulation; **b** Cartoons of four DONs and their key geometric parameters. One grid was 50 nm. “L” was the length of rod or the ring’s perimeter; “**d**” was the “cross-section “of the bundled DNA helices into a honeycomb lattice. The triangle was composed of only 1 bundled DNA helices, thus d was 2 nm, and the rod ring and octahedron were made of 3 bundled DNA helices, so d was 6 nm; “D” was the diameter of ring’s outer circle; “a” was the length of each side of an equilateral triangle; “h” was the distance between the two farthest points in the octahedron; the vertex number was defined as the number of points on an object where the sides or edges of the structure were meet. Note, the size of DONs was marked by the letter of L, D, a and h, respectively. The vertex number of nanorod was 1 owing to the rod bending into a V-shape; **c** Calculation of key geometric parameters of DONs, including the ASA, EV, AR, size (conventional characteristics in yellow highlight) and vertex number (new characteristics in blue highlight). The calculation of theoretical size referred to Suppl. Mater. Excel S1

Using caDNAno and oxDNA2, four types of DONs were designed and simulated as follow: a one-dimensional (1D)-nanorod, a 1D- ring formed through connecting the long nanorod’s one end to another end (1D), a 2D-planar equilateral triangle (2D) and a 3D-octahedron (Fig. 2). Figure 2a shows the results of relaxation of four DONs (rod, ring, triangle, rod and octahedron). Before MD simulation, the rod, ring and triangle were initially designed in caDNAno as two-dimensional sheets, and after simulation, the ring and triangular configurations were formed via bending a long rod for end-to-end connection, and via convergence of three trapezoidal domains, respectively, by adding additional staple DNA with modified backbone potential forces. No structural change was observed in the 1D- nanorod. The octahedron was composed of 12 equal sides in caDNAno design, and after the relaxation, a 3D octahedron configuration was constructed via stretch-folding the *ss*DNA scaffold using staples *ss*DNA. All of four DONs were folded by anchoring staples to the scaffold through the molecular forces based on the Watson–Crick base pairing, where the base side lengths of each DONs were interwoven to form a more stable ortho-hexagonal honeycomb structure by arranging the 6 *ss*DNA parallel to each other. The octahedron and triangle were also designed with a reduction of some of the staples in location of vertext point to reduce the structural stress of the DONs due to their distinct structural apexes.

Individual DONs were also characterized by distinct geometric parameters, which were defined and calculated based on the design of DONs (Fig. 2b, c). The sizes of individual DONs was estimated based on the contour length of DNA polymer. The contour length is the distance between two adjacent bases is measured as 0.34 nm [41]. Considering the total 7249 n.t. in the *ss*DNA M13mp18 scaffold and its folding route design (Supple. Mater. Figure S1andExcel S1), all four DONs were at the nanoscale with the sizes of 410 nm for the rod, 131 nm for the ring, 126 nm for the triangle and 44.2 nm for the octahedron (Fig. 2b). The conventional geometric parameters (ASA, EV, compactness, AR) of individual DONs was further calculated by defining the diameters, perimeter, length and the distance in individual DONs, and each geometric characteristics of four DONs increased in the following order (Fig. 2c in yellow highlight): triangle < ring < octahedron < rod for ASA, rod < triangle < octahedron < ring for EV, ring < octahedron < triangle < rod for compactness, and octahedron < ring < triangle < rod for AR. Despite of increased structural complexity from 1 to 3D, all designed DONs had the same controlled mass of 4780 kDa, enabling direct comparison of geometric parameters (ASA, EV, compactness, AR, size and vertex number) among different DONs.

The vertices number was a new geometric characteristic (Fig. 2c in blue highlight). The vertex number of DONs was defined as the number of points where the sides or edges of nanostructure were meet, and it increased as their nanostructure complexity increased from 1 to 3D. The 2D-planar triangle and 3D-octahedron were characterized of 3 and 6 fixed folding points, respectively. In contrast, the 1D-nanorod had a fixed bending point during self-assembly, thus giving the vertex number as 1, while the 1D-ring had the vertex number of 0 as it did not form any folding point of its edge in its structure. From the point of view of DONs design, the number of vertices in each DONs had presented the folding of the *ss*DNA scaffold into varying numbers of strands (i.e., edges): 3 in the triangle, 1 in the rod, none in the ring, and 12 in the octahedron (Fig. 2a). DNA bending rigid is closely related to DNA persistence length, and by consensus, its value is approximately 150 bp or 50 nm [42–44]. The more vertices present in one DONs, the shorter length of each DNA edge was close to the DNA persistence length. This is consistent with geometric definition about rigidity, for which is the ability of a material or structure to resist elastic deformation when subjected to a force and is an indication of the extent of difficulty in elastic deformation of the material or structure [45, 46]. As a result, the octahedron and the triangle were non-transformable structures in space compared to the rod and ring with some degrees of freedom for changes in spatial structures.

### Geometric characterization of ensembled DONs

**Fig. 3:**
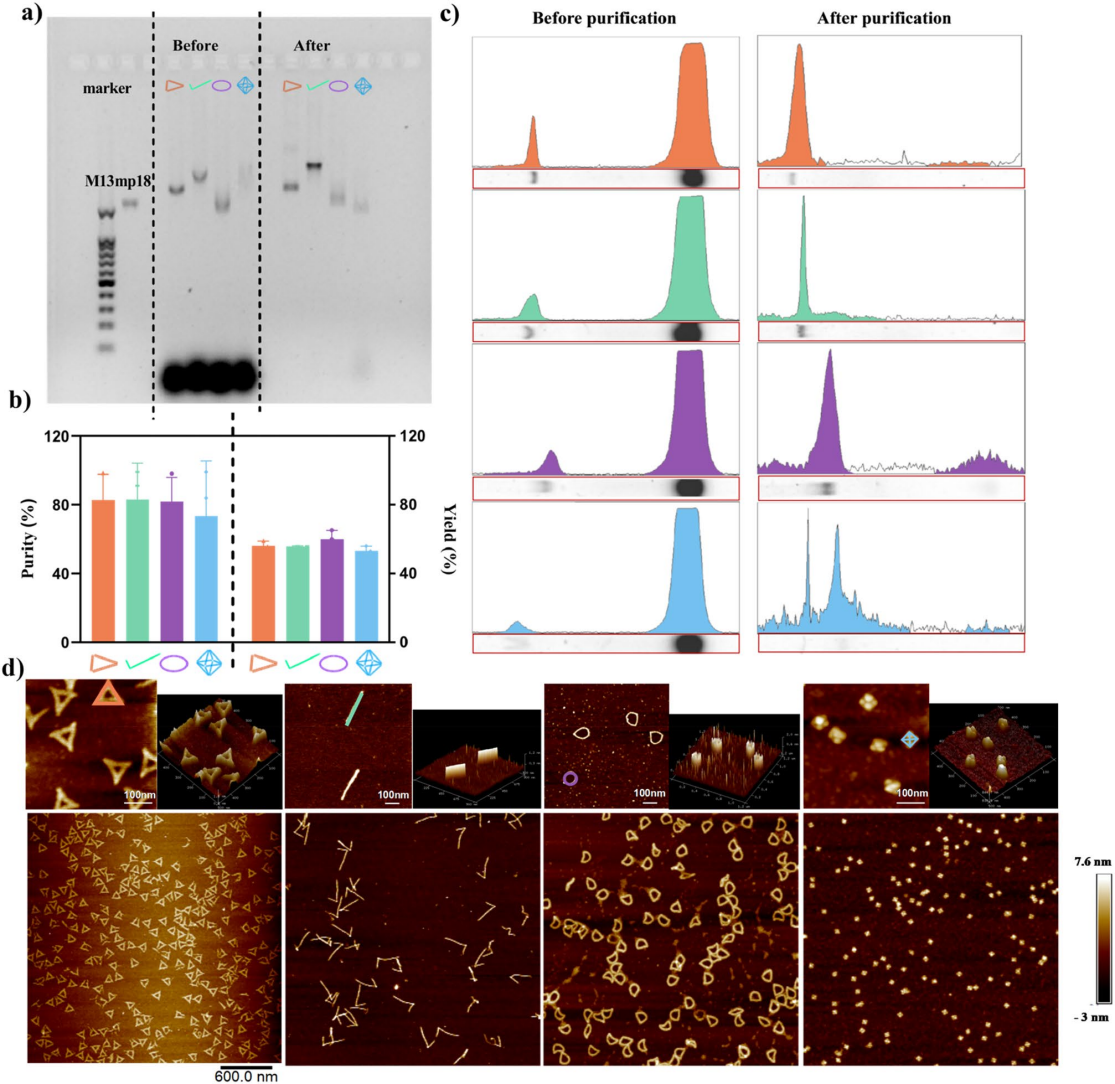
Purification and structure characterization of four fabricated DONs. **a** AGE of assembled DNA-origami nanostructures before and after purification. Lanes (from left to right): DNA Ladder (100–4100 bp), M13mp18 ssDNA (7249 n.t.), the ring, rod, triangle and octahedron; **b** AGE band profiles before and after purification. The OD values of the samples and the short strand of excess staples before and after purification were quantified by image J; **c** Purity% and Yield% of fabricated DNA nanostructures. The theoretical yield referred to Suppl. Mater. Excel S2. Individual samples (6 mL) were loaded onto 1.2% agarose gel and run in 1 × TAE buffer with 12.5 mM MgCl_2_ at 100 V for 1 h; **d** AFM images of four purified DONs. From left to right: 2D images of triangles, rods, ring and octahedron. Each DONs had the scale bars of 600 nm and 100 nm images and a 3D image. DONs were marked in color in the 2D images (scale bar 100 nm). All the samples were diluted 4 × with 1 × TAE (12.5 mM Mg^2+^) and loaded onto the PLL coating mica. All samples were in triplicates

Experimentally prepared DONs were evaluated based on their purity%, yield%, intactness, structure and stability before cellular uptake study. Ultracentrifugation can effectively remove excess staples from the DNA folding reaction (Fig. 3a–c, and Suppl. Mater. S4−5). The purity% after ultracentrifugation can reach above 73% for all DONs, and the yield% of four DONs were similar, ranging from 54 to 59% owing to similar folding kinetics and annealing environment. To reduce the influence of the design of the bracket route on the final form of the DONs, Seam design was adopted for all four DONs. Seam design is the most classic design and its folding process was close to the zipper behavior starting from both ends instead of one end [47]. Compared to other 2D- and 3D- DONs, the 1D-nanorod had the least design complexity that did not require a large spatial scale of folding and joining, thus reducing the time required for Brownian dynamics at the molecular level. As a result, the ring had slightly higher yield% (i.e., 59%) than the other three structures. It is worth noting that the current yield% is not high compared to other DNA-origami studies reaching 50–90% [48–50], which may be attributed to the set-up of annealing temperature and the ultracentrifugation process for purification [51].

Using AFM, four DONs were uniformly distributed in the 3 μm × 3 μm field of the images (Fig. 3d), and their dimensions and nanostructures were consistent with the theoretically calculated sizes (Fig. 2b, c). Compared to non-fixed morphology in the ring and rod, uniform and well-defined DONs for the triangles and octahedra in AFM images provided evidences for rigid structure associated with the vertex number. The 2D-triangular DONs displayed a perfect triangular morphology with three sharp vertices. The top- and side-view AFM image of octahedra showed the rigid half-structure of the tetrahedron characterized of a regular square or prismatic morphology in the 2D-AFM image. On the other hand, AFM images of the circle and rod revealed good maintenance of structural integrity with some degree of freedom for morphological changes. For example, 30% of nanorod DONs showed a V-bending into a hook-like structure with one vertex. This may be due to the fact that the length of our design exceeds the strength that the structure can withstand, and thus most of the rod structure had a rigid apex. and the formation of this node can shorten the length of a single side of the structure, thus avoiding the secondary appearance of the second folding point.

### Stability and fluorescent calibration of Cy5-labelled DONs

**Fig. 4:**
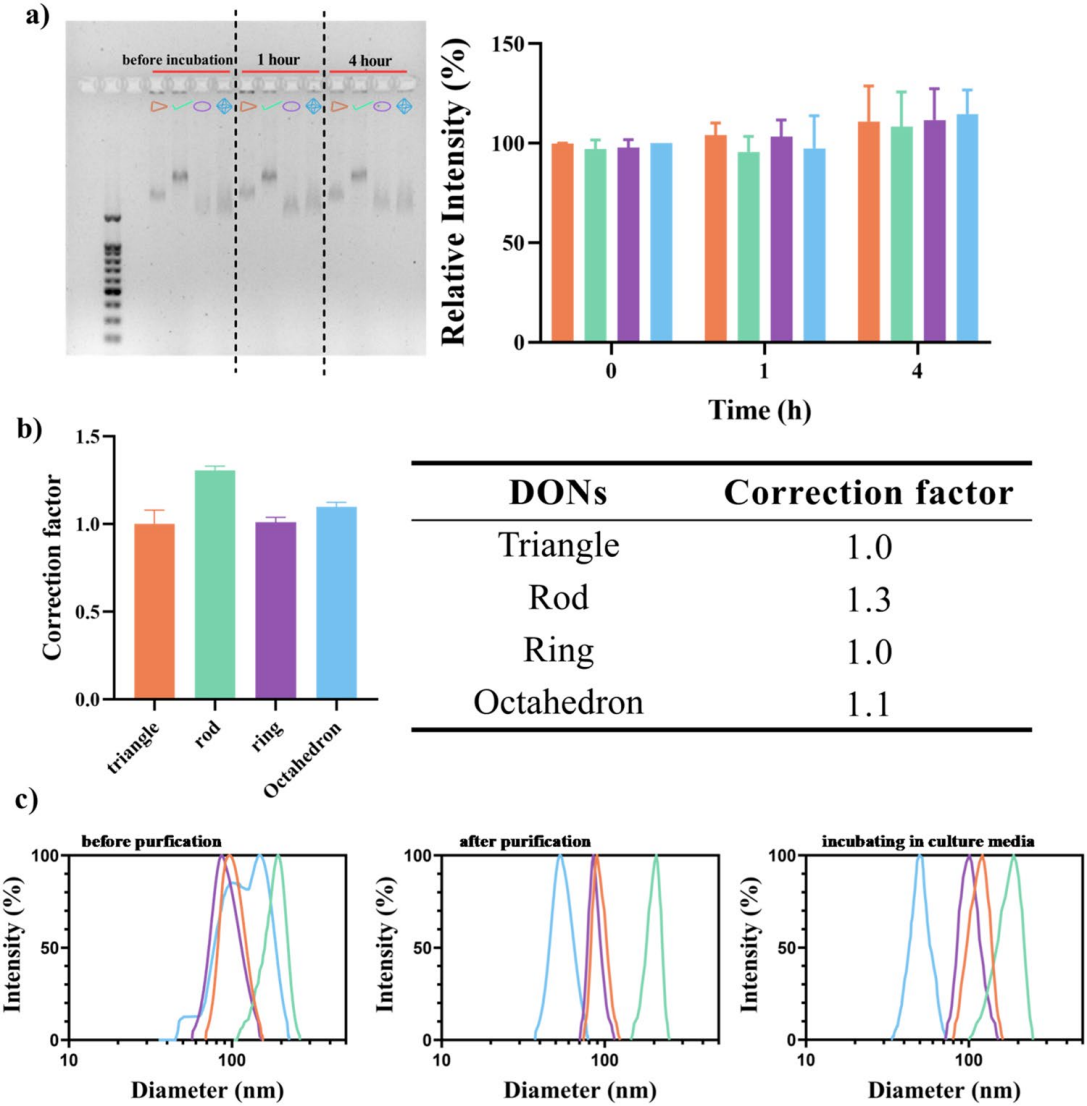
Integrity and Cy5 fluorescence calibration of four DONs. **a** AGE showing DONs incubated in MEM supplemented with 10% FBS at 37 °C for 1 h and 4 h. The gel band intensity was quantified using the image J, and the relative intensity percent (%) for each structure was calculated by comparing the intensity at pre-designated time (1 h or 4 h) with that before incubation. Each color coded for triangle (orange), rod (green), ring (purple) and octahedron (blue); **b** Correction factor of each DONs. Triangle was used as the internal standard for determining correction factor of other nanostructures (Suppl. Mater. S8).** c** The intensity-weighted distribution of Cy5 labelled DONs hydrodynamic diameters under various preparation conditions. From left to right, before and after purification and at while incubating in MEM supplemented with 10% FBS at 37 °C for 4 h. All samples were in triplicates

Structure integrity of Cy5-labeled DONs via base-pairing has been widely demonstrated in the cellular environment compared to linear DNA [52, 53]. Under our experimental conditions, each of four DONs was incubated in the standard cell culture medium supplemented with 10% FBS at 37 °C for 1 h and 4 h. We did not observe any decrease in OD values of the electrophoresis bands of the DONs compared to ones before incubation (0 h) (Fig. 4a), demonstrating the resistance of the DONs to hydrolysis by nucleases. To ensure that the cellular measurements among DONs were quantitative, Cy5-fluorescence of DONs was normalized by determining the correction factor of each DONs based on the triangular DONs as an internal standard in Fig. 4b. All four DONs had correction factor near one, indicating three Cy5-fluorophore in the staples were effectively incorporated into each DONs. The sizes of Cy5-labelled DONs were also measured under various preparation conditions using DLS. Compared to purified samples, the diameter (outer dimension) of DONs incubated in cell culture median supplemented with 10% FBS were similar, indicating no aggregation of DONs (Fig. 4c). It is worth noting that octahedral DONs markedly differed in the diameter distributions as evidenced by excessive aggregation (i.e., large size) of octahedron before purification presumably due to the excess amount of staple ssDNA (Fig. 4c). This could explain the altered band position of octahedron before and after purification in Fig. 3a. The DONs stability and normalization of number of incorporated Cy5 fluorophores in each DONs warrant further cellular uptake study of DONs.

### Cell internalization of DONs in hLECs

**Fig. 5:**
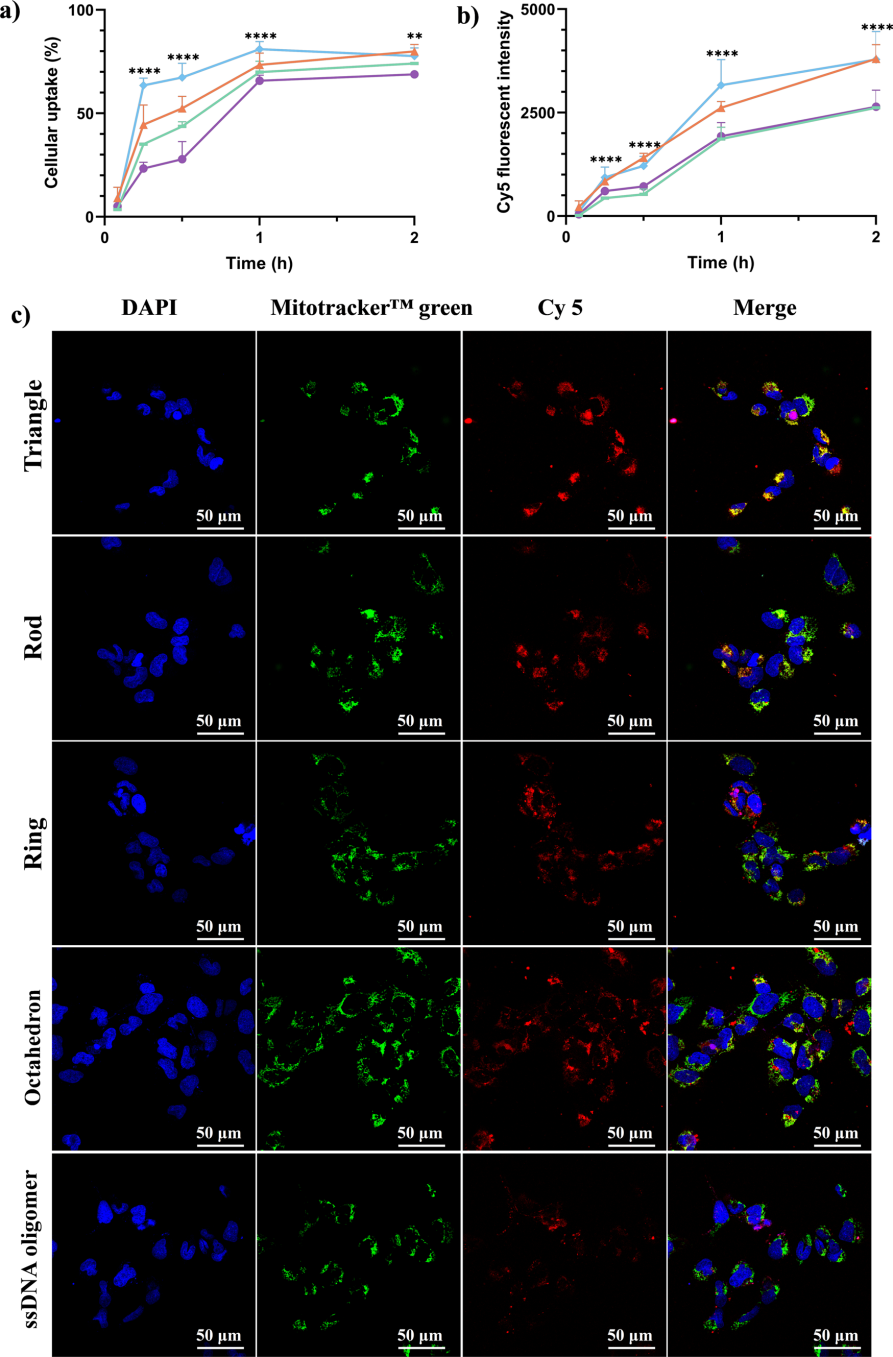
Cellular uptake and biodistribution of four DONs in *h*LECs HLE-B3. **a** Cellular uptake (%) of DONs in HLE B-3 cells; **b** Cy5 fluorescent intensity of gated cell population in each DNA nanostructure treatment group. The number of HLE B-3 cells acquired was 30,000. The results were analyzed by the FlowJo_v10.8.1 software. Different colored points & connecting lines represented the triangle (orange), rod (green), ring (purple) and octahedron (blue); **c** CLSM images of intracellular localization of DONs in HLE-B3 cells. Cy5-labelled ssDNA oligomers were used as a control. Nucleus were in Blue (DAPI) detect using 405 nm Blue channel, mitochondria were labeled in green (Mitotracker green) detected using 488 nm green channel, and DNA nanostructures were in red (Cy5) detected using 648 nm red channel. For both flow cytometry and CLSM experiments, HLE B-3 cells were incubated with individual DONs at 0.4 nM for 2 h. All samples were repeated three times. The CLSM images were provided with 60× magnification and 10,241,024× pixels in size

To determine the cellular entry capacity of four DONs (the triangle, rod, ring and octahedron), their uptake and biodistribution in *h*LECs HLEB-3 were evaluated by a flow cytometry and CLSM. Firstly, all four DONs exhibited time-depend cellular uptake (Fig. 5a, b, Suppl. Mater. S8). At an early time of 5 min, cellular uptake percent (%) (Fig. 5a) and Cy5 fluorescent intensity (Fig. 5b) was similar among the four DONs. At 15 min and 30 min, four DONs exhibited markedly difference in cell entry capacity, for which the rigid DONs (triangle and octahedron) had 1.7-fold and 2.1-fold higher cellular uptake% and total Cy5 fluorescent intensity, respectively, than that of the rod and ring. At the 1 h and 2 h, both cellular uptake% and accumulation reached the plateau. In the flow cytometry analysis, cellular uptake% was obtained by comparing to total number of *h*LECs, thus providing information on the fraction of cells taking up individual DONs, while Cy5 fluorescent intensity showed the total amount of DONs accumulated in *h*LECs.

Intracellular distributions to the mitochondria and nucleus in *h*LECs HLE B-3 2 h after treatment with DONs were imaged using CLSM (Fig. 5c). Compared to Cy5-labelled ssDNA staples (control) with weak Cy5-fluorescent signal, CLSM images of all four DONs (triangles, rods, rings, octahedron) showed bright red fluorescence, indicating enhanced cellular uptake. More importantly, DONs with distinct geometries showed different extent of cellular uptake as evidenced by stronger Cy5 fluorescent intensity (red) for both triangle and octahedron compared to the rod and ring. This is consistent with the flow cytometry analysis that the rigid DONs (i.e., triangle and octahedron) uptake in *h*LECs were more than non-rigid DONs (i.e., the rod and ring). The co-localization of four DONs was not uniform throughout the cells with a large number of DONs present in the close vicinity of the mitochondria shown as yellowish color merged by the red Cy5 fluorophore and the green mitochondrial dye. This is especially evident for the triangles and octahedra. In some cells, the DONs entered the nucleus, which appeared in purple color merged by the blue nucleus dye and the red Cy5 fluorophore. It is worth noting that the nuclease degradation of DONs in biological systems could result in the release of free cyanine dyes that may potentially give the false signal using the fluorescent dye labeling technique [52]. Although we did not observe the degradation over 4 h in our experimental conditions (Fig. 4), it is always cautionary to include the proper dye control with the understanding of their physical and fluorescent properties [54, 55].

### Geometric correlations with DONs uptake in hLECs

**Fig. 6:**
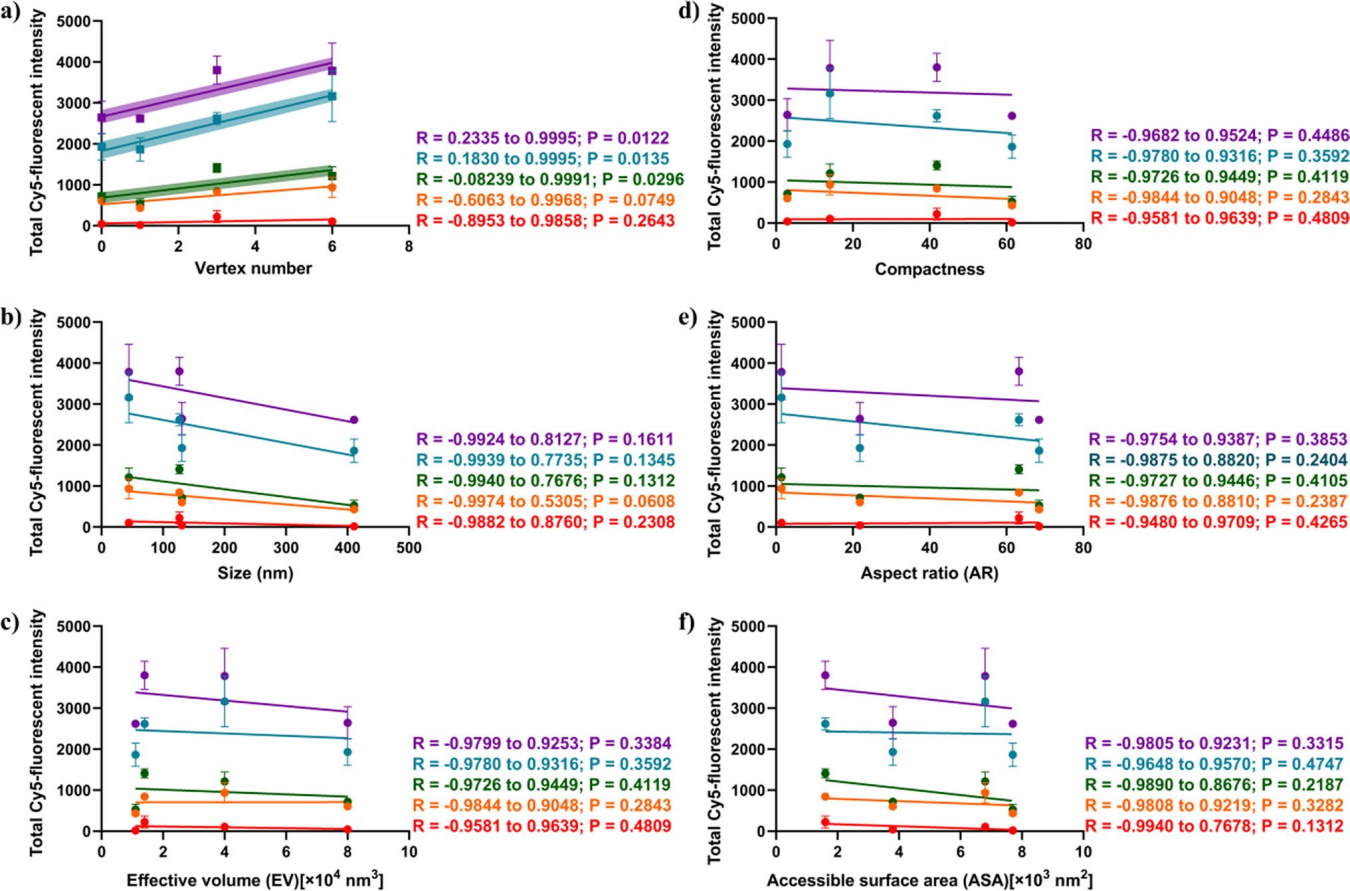
Pearson correlation analysis of geometric parameters of four DONs and total Cy5 fluorescent intensity over time in hLECs. Correlation plots of **a**Vertex number, **b** Size, **c** Effective volume, **d** Compactness, **e** Aspect ratio, and **f** Accessible surface area. Pearson with one-tailed analysis was applied. Groups with statistical significance were highlighted. Different colors coded for fluorescent intensity at pre-determined incubation time points of DONs at 5 min (red), 15 min (orange), 30 min (green), 1 h (blue), and 2 h (purple). Each data point represented one DNA nanostructure with n = 3. R is the *Pearson’s* correlation coefficient (95% confidence interval), for which R between 0 and 1, at 0 and between 0 and − 1 measures positive-, no- and negative correlations, respectively. *P* < 0.05 means that the two variables was significantly correlated

**Fig. 7:**
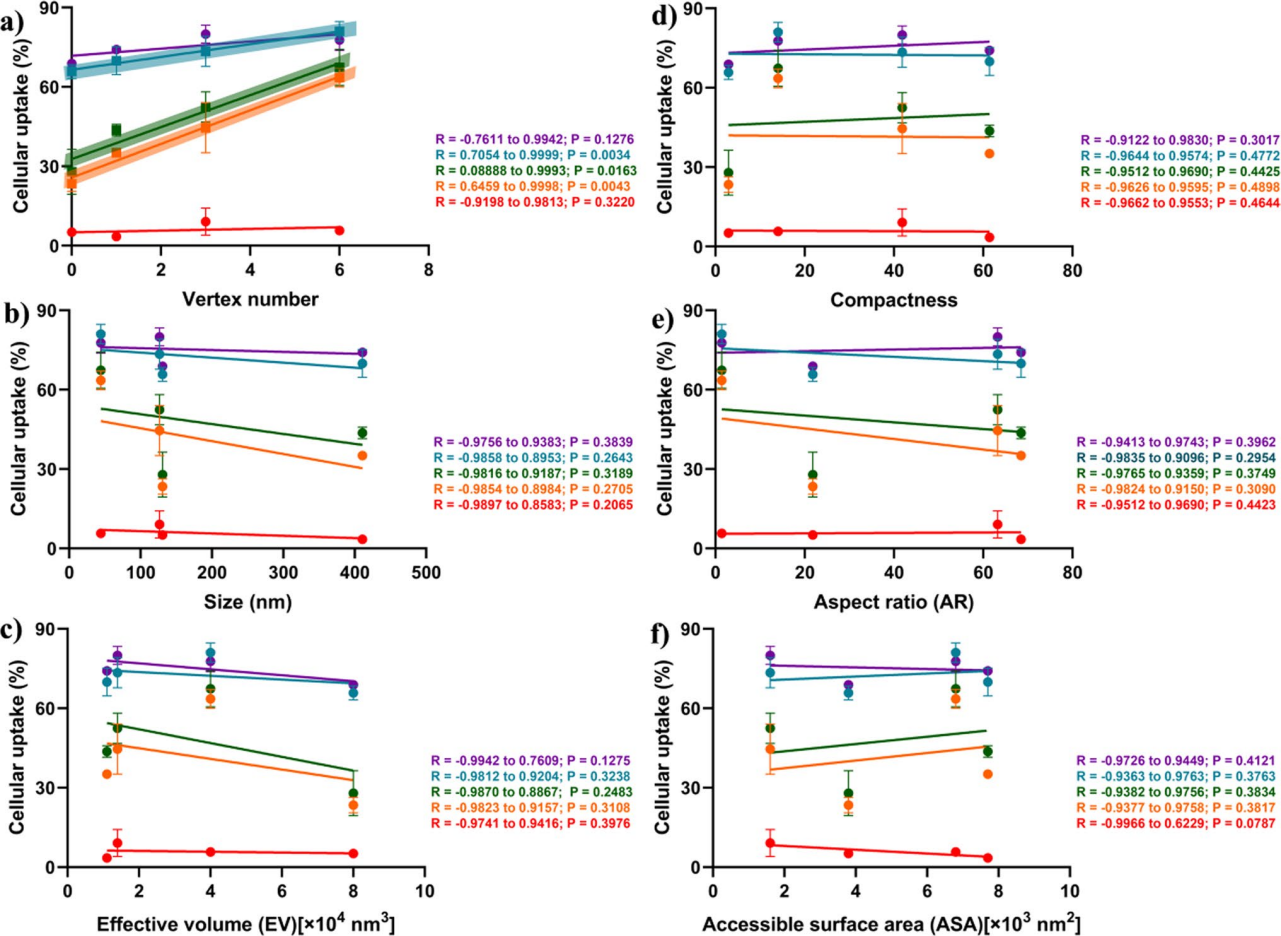
Pearson correlation analysis of geometrical parameters of four DONs and cellular uptake (%) over time in *h*LECs. Correlation plots of **a** Vertex number, **b** Size, **c** Effective volume, **d** Compactness, **e** Aspect ratio, and **f** Accessible surface area. Pearson with one-tailed analysis was applied. Groups with significance are distinguished by highlight. Different colors coded for cellular uptake (%) at pre-determined DNA nanostructure incubation time points at 5 min (red), 15 min (orange), 30 min (green), 1 h (blue), and 2 h (purple). Each data point represented a DNA structure with n = 3. R is the Pearson’s correlation coefficient (95% confidence interval), for which R between 0 and 1, at 0 and between 0 and −1 measures positive-, no- and negative correlations, respectively. *P* < 0.05 means that the two variables are significantly correlated

*Pearson* correlation coefficient (*R*) was performed to analyze the relationship between DONs geometric characteristics and their uptake in *h*LECs determined by the flow cytometry. The two-sets of correlations were analyzed: (1) between geometric characteristics and total Cy5-fluorescent intensity of DONs (Fig. 6), and (2) between geometric characteristics and cellular uptake% of DONs (Fig. 7). The vertex number of DONs had significant and positive correlations at 30min, 1 h and 2h for total Cy5-fluorescence, and at 15min, 30min, and 1h for cellular uptake%, respectively, with *R* values between 0.71 and 0.99 close to 1 and *p* < 0.05, meaning that increasing the vertex number in DONs will increase DONs uptake in *h*LECs. It is noting that total Cy5-fluorescent intensity of DONs evaluated the number of DONs accumulated in HLE B-3 cells (i.e., how many), while cellular uptake% suggested the cellular uptake rate of DONs (i.e., how fast). Accordingly, during the process of cellular internalization, the vertex number in DONs had an impact on the DONs accumulation in hLECs at the late time-to-plateau stage, while on the cellular uptake of particles at the early time-before reach the plateau. Other geometric parameters (ASA, EV, AR, compactness and size) did not show significant correlations with cell entry indicated by a wide *R* values ranging from − 0.99 to 0.99 and *p* > 0.05. Because only four DONs were explored, the limited data sets in *Pearson*’s correlations may not reflect the actual impact of other geometric traits on the uptake efficiency.

Despite of no significant correlations observed between cellular entry and geometric characteristics (e.g., sizes and AR) in our DONs design, several studies have shown that sizes and geometric forms of particles can dictate the process of cellular internalization. Bastings et al. screened the uptake of 9 DONs library in endothelial, epithelial and immune cell lines and found that a linear correlation between DONs compactness and uptake efficiency, and smaller AR between 1 and 3 were favored for uptake in bone-marrow-derived dendritic cells [30]. Another study in various lung cancer cell lines found that the large DONs of solid rod and hollow tetrahedra exhibited higher cellular uptake efficiency than small DONs of the same shapes (i.e., 127 nm vs. 32 nm in length for rods and 47 nm vs. 11 nm in length for tetrahedra), and scavenger receptors were shown to mediate the endocytosis of small and larger DONs [31]. It is worth mentioning that geometric parameters of particles, such as AR, does not obey the linear correlations; rather, it follows non-monotonic behavior. Studies of 1D- 2D- and 3D- nanorods have shown that distinct entry modes: AR of 3D-nanorod dictated the angel of entry into cells, where AR > 2 exhibited “laying down to stand up” mode and AR = 1 and 1.5 exhibited direct particle rotation or slight tilt angle [56], whereas 1D- and 2D- nanorods without AR typically had two basic modes for cell entry: perpendicular tip- and parallel surface adhering entries, depending on the cell membrane tension [57]. This may explain the lack of linear correlations observed between geometric characteristics (e.g., AR) and cellular entry.

The inherent physiochemical properties of nanoparticles and cell membrane, such as the size, shape, stiffness, surface chemistry, and elasticity of cell membrane, affect possible choices (e.g., endocytosis vs. phagocytosis), rate and amount of particle internalization into cells [58, 59]. As illustrated in Fig. 2, the number of vertices in DONs form multiple sharp corners (e.g., triangle and octahedron) that interact at the cell membrane interface of *h*LECs HLE B-3 cells. A study of DONs internalization in HeLa cells found that tetrahedral DONs approached cells with their corners (or the vertex point) for cellular engulfment rather than “face attack” (or spreading), and such cellular uptake model reduced the electrostatic repulsion between negative charged DONs and cell membrane [60, 61]. The DNA-folding frames with more vertices may be structurally more rigid owing to the presence of DNA persistent length of DONs [42–44] as discussed in “3.1 Geometric Design of Four DONs”. Compared to “Soft” nanoparticles with higher extent of spreading on the cell membrane, spiky and rigid NPs require less bending energy to be endocytosed cross the cell membrane [62–64].The frames of the triangular and octahedral DONs have been described as “rigidness” to enhance uptake of cancer cells [65, 66]. In particular, compared to conventional spherical polymer-based micelles without any sharp corner, 2D-triangular and 3D-tubular DONs reversed drug-resistance in MCF-7 breast cancer cells by enhanced drug uptake to the site of drug action (i.e., nucleus), suggesting the shape-dependent cell uptake [67].

### DONs localization at sub-cellular organelles in hLECs

**Fig. 8:**
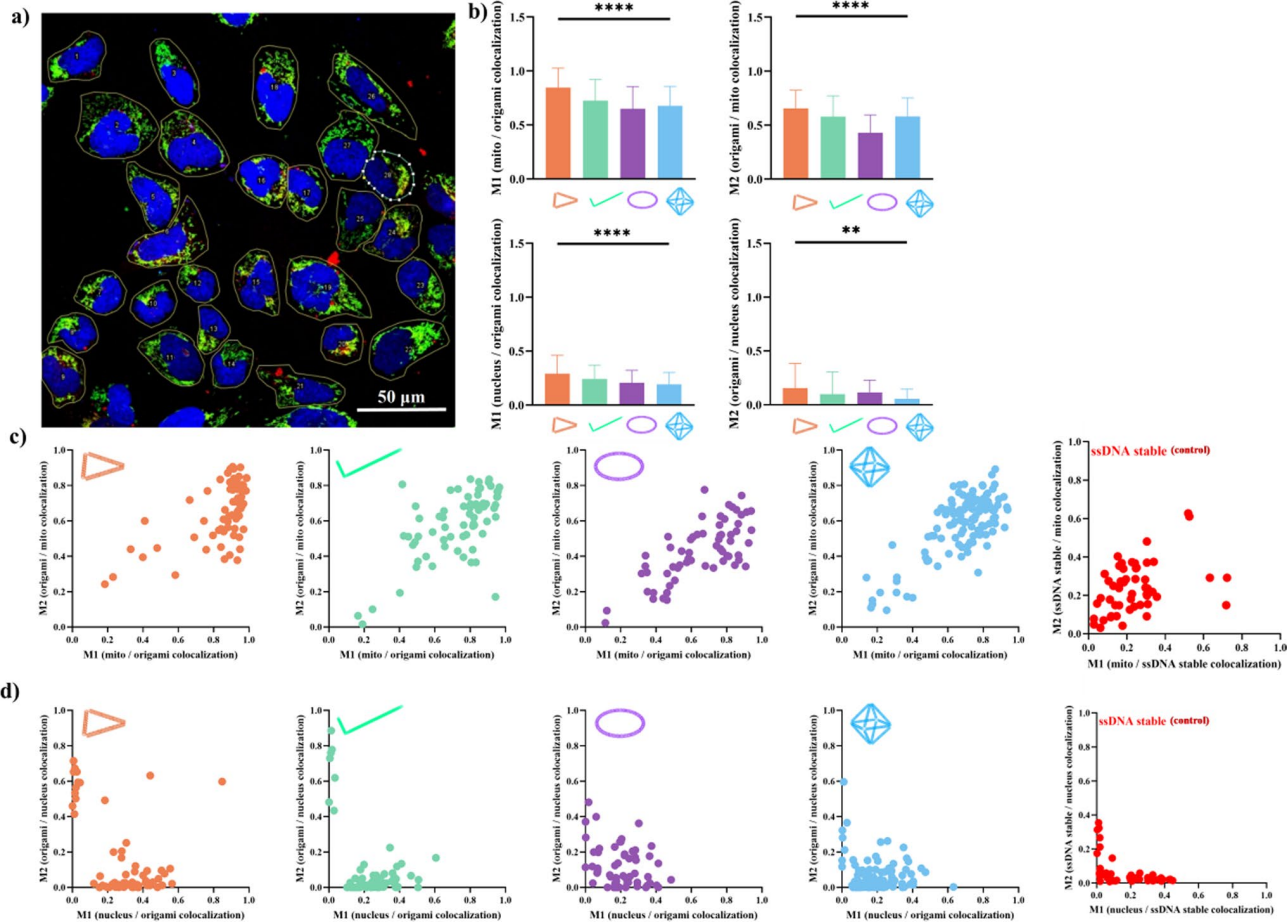
MCC analysis of four DONs co-localization at mitochondrial and nucleus in CLSM images. **a** Illustration of co-localization in confocal images using Image J2 software. HLE-B3 cells was selected according to the intactness and morphology of nucleus and a good staining of mitochondria, and then individually circled using the color 2 module to analyze MCC. The colors of blue, green and red in the image represented the nucleus, mitochondria, and DONs, respectively. Of the four structures triangles, straight rods, rings and octahedrons, 67, 68, 62 and 117 cells were selected for MCC analysis, respectively; **b** Quantification of the MCC M1 [fraction of mitochondria (top) or nucleus (bottom) in colocalization with origami shapes) and M2 [fraction of origami shapes in colocalization with mitochondria (top) and nucleus (bottom)]; **c** M1 vs. M2 MCC distribution for co-localization of DONs and mitochondria; **d** M1 vs. M2 MCC distribution for co-localization of DONs and nucleus. Data were mean ± sd. *P < 0.05; **P < 0.01; ***P < 0.001; ****P < 0.0001. Ordinary One-way ANOVA (GraphPad, un-paired, Gaussian distribution) with multiple comparisons was used to compare means among groups

MCC was applied to selected *h*LECs in the single confocal sliced images to quantitatively analyze the degree of fluorescence co-localization between Cy5 and DAPI (at nucleus) as well as Cy5 and Mitotracker Green FM (at mitochondria) (Fig. 8). MCC is a statistical method of measuring the degree of co-localization of dual-color confocal images, and its co-localization coefficients M1 and M2 provide quantitative information about the position relation between Cy5-labeled DONs and intracellular compartments (i.e., nucleus and mitochondria). Significant difference among four DONs were observed in terms of both M1 and M2 at mitochondria and nucleus. Values of M1 and M2 (less than 0.5) at nucleus were lower than mitochondria (Fig. 8b), suggesting the degree of Cy5-labeled DONs positioned at nucleus were not high. It is worth noting that triangular DONs had the highest M1 and M2 of co-localization with mitochondria than other DONs (Suppl. Mater. S10). The MCC-plots of M1 vs. M2 of individual *h*LECs at both mitochondria and nucleus showed a high degree of co-localization of four DONs at mitochondria as evidenced by distribution of analyzed cells in the upper right region of the plots, whereas in nucleus, most of M1 vs. M2 of hLECs were distributed in the lower left corner, indicating weak co-localization of four DONs at nucleus (Fig. 8c, d). In contrast, the degree of co-localization of the Cy5-labelled ssDNA staples with either mitochondria or the nucleus was poor, further suggesting the ensembled DONs was critical for cellular uptake. Pearson’s correlation coefficient (PCC) analysis showed the similar trend of co-localization of DONs at mitochondria and nuclei with DONs, as detailed in the Suppl. Mater. S9, where PCC was positive for mitochondrial co-localization and negative for nucleus [68].

Targeting cells by designed nanostructures can empower cell-based therapy. The present study provided quantitative information of the co-localization degree of DONs at mitochondria and nucleus. but cell entry pathways of DONs are still under intensive investigation. Using high-resolution transmission electron microscopy, long DONs nanorods (127 nm in length with a cross-section of 8 nm) underwent four distinct stages of cellular internalization, including binding with membrane, initiation of invagination, transport to early endosome and transport to late endosome/lysosome [31]. One study revealed the transport of tetrahedron DONs in the cytoplasm were microtubule dependent with assistance of molecular motors, such as kinesin and dynein [69]. Many studies have functionalized DONs with various molecular moieties to actively target cells, to escape from endo-/lysosomal sequestration and to import into cellular compartments, such as nucleus. For example, DONs were conjugated with tumor-associated overexpressing proteins (e.g., thrombin and mucin) to target cancer cells [70, 71]. DONs enter the cell through various endocytosis pathways, such as clathrin- and caveolae mediated and scavenger receptors, depending on their interaction with the cellular surface [72]. A tetrahedral DONs were functionalized with nucleus-targeting signaling peptides to direct their escape from the lysosomes and entry into the nuclei) [69]. Deformation of cell membrane was also attempted to facilitate DONs into cytosolic compartment via transiently enlarged membrane holes using the microfluidic device [73]. On the other hand, endosomal acidic pH was hypothesized to facilitate the drug release from DONs for enhanced cytotoxicity in drug-resistant cancer cells [74]. To reach target cellular compartments, for example, nanorods (30 nm) conjugated with anti- RNA-Pol II (a nuclear factor) antibody was cytosolically bound to RNA-Pol II and further imported into live-cell nuclei [32]. Another study utilized the characteristics of specific localization of lipid moiety (e.g., cholesterol and alkyl chains) anchored DONs into lipid bilayer regions, and further built DNA nanocages bearing a double-tailed C18 lipidic chain to improve targeting to mitochondrial membrane, resulting in increased anticancer drug doxorubicin accumulation at mithochondria for enhanced cytotoxicity in cancer cells [75, 76]. All of those studies have demonstrated potential application of intracellular delivery using nanostructures prepared by DNA-origami technique.

## Conclusion

In conclusion, four non-spherical particles (rod, ring, triangle and octahedron) with defined geometric parameters of ASA, EV, AR, size, vertex number and compactness were fabricated by using computer-aided DNA-origami design. Prepared DONs were in high quality as evidenced by their good yield and structural intactness in the cell culture media. In *h*LECs, four DONs exhibited differential cellular entry with both triangles and octahedra showing higher cell uptake% and accumulation than that of the ring and rod. *Pearson’s* correlation further suggested the positive correlations between vertex numbers in DONs and fluorescent intensity of cellular uptake. DONs showed different degrees of co-localizations at mitochondria and nucleus, and triangles had the highest degree of mitochondrial localization among other DONs. This study sheds light on the impact of nanostructures on cell targeting ability and provides insights into the structure-modulating design of nanomedicine for ocular therapy.

## Supplementary Information

Additional file 1

Additional file 2

## Data Availability

Data is provided within the manuscript or supplementary information files.
