# Supplementary material for: Correlation of precisely fabricated geometric characteristics of DNA-origami nanostructures with their cellular entry in human lens epithelial cells
Source: Discov Nano. 2025 Jan 22;20(1):13. doi: 10.1186/s11671-025-04188-9 (PMC11754578; doi:10.1186/s11671-025-04188-9)
Supplement: Supplementary file 1 — Additional file 1 [file 11671_2025_4188_MOESM1_ESM.docx]

Supplementary Materials

Correlation of Precisely Fabricated Geometric Characteristics of DNA-Origami Nanostructures with Their Cellular Entry in Human Lens Epithelial Cells

Yexuan Guo^1#^, Tianze Xiong^1#^, Hong Yan^1,2*^, Rui Xue Zhang^1*^

^1^ Institute of Medical Research, Northwestern Polytechnical University, 127 West Youyi Road, Xi’an, Shaanxi 710072, China

^2^ Xi’an People’s Hospital (Xi’an Fourth Hospital), Shaanxi Eye Hospital, 21 Jiefang Road, Xi’an, Shaanxi 710004, China

^#^ These authors contributed equally to this work;

^*^Corresponding authors: Rui Xue Zhang ([zhangruixue@nwpu.edu.cn](mailto:zhangruixue@nwpu.edu.cn)); Hong Yan ([yan2128ts@med.nwu.edu.cn](mailto:yan2128ts@med.nwu.edu.cn))

S1. Linear ssDNA scaffold M13mp18 sequence

AATGCTACTACTATTAGTAGAATTGATGCCACCTTTTCAGCTCGCGCCCCAAATGAAAATATAGCTAAACAGGTTATTGACCATTTGCGAAATGTATCTAATGGTCAAACTAAATCTACTCGTTCGCAGAATTGGGAATCAACTGTTATATGGAATGAAACTTCCAGACACCGTACTTTAGTTGCATATTTAAAACATGTTGAGCTACAGCATTATATTCAGCAATTAAGCTCTAAGCCATCCGCAAAAATGACCTCTTATCAAAAGGAGCAATTAAAGGTACTCTCTAATCCTGACCTGTTGGAGTTTGCTTCCGGTCTGGTTCGCTTTGAAGCTCGAATTAAAACGCGATATTTGAAGTCTTTCGGGCTTCCTCTTAATCTTTTTGATGCAATCCGCTTTGCTTCTGACTATAATAGTCAGGGTAAAGACCTGATTTTTGATTTATGGTCATTCTCGTTTTCTGAACTGTTTAAAGCATTTGAGGGGGATTCAATGAATATTTATGACGATTCCGCAGTATTGGACGCTATCCAGTCTAAACATTTTACTATTACCCCCTCTGGCAAAACTTCTTTTGCAAAAGCCTCTCGCTATTTTGGTTTTTATCGTCGTCTGGTAAACGAGGGTTATGATAGTGTTGCTCTTACTATGCCTCGTAATTCCTTTTGGCGTTATGTATCTGCATTAGTTGAATGTGGTATTCCTAAATCTCAACTGATGAATCTTTCTACCTGTAATAATGTTGTTCCGTTAGTTCGTTTTATTAACGTAGATTTTTCTTCCCAACGTCCTGACTGGTATAATGAGCCAGTTCTTAAAATCGCATAAGGTAATTCACAATGATTAAAGTTGAAATTAAACCATCTCAAGCCCAATTTACTACTCGTTCTGGTGTTTCTCGTCAGGGCAAGCCTTATTCACTGAATGAGCAGCTTTGTTACGTTGATTTGGGTAATGAATATCCGGTTCTTGTCAAGATTACTCTTGATGAAGGTCAGCCAGCCTATGCGCCTGGTCTGTACACCGTTCATCTGTCC

TCTTTCAAAGTTGGTCAGTTCGGTTCCCTTATGATTGACCGTCTGCGCCTCGTTCCGGCTAAGTAACATGGAGCAGGTCGCGGATTTCGACACAATTTATCAGGCGATGATACAAATCTCCGTTGTACTTTGTTTCGCGCTTGGTATAATCGCTGGGGGTCAAAGATGAGTGTTTTAGTGTATTCTTTTGCCTCTTTCGTTTTAGGTTGGTGCCTTCGTAGTGGCATTACGTATTTTACCCGTTTAATGGAAACTTCCTCATGAAAAAGTCTTTAGTCCTCAAAGCCTCTGTAGCCGTTGCTACCCTCGTTCCGATGCTGTCTTTCGCTGCTGAGGGTGACGATCCCGCAAAAGCGGCCTTTAACTCCCTGCAAGCCTCAGCGACCGAATATATCGGTTATGCGTGGGCGATGGTTGTTGTCATTGTCGGCGCAACTATCGGTATCAAGCTGTTTAAGAAATTCACCTCGAAAGCAAGCTGATAAACCGATACAATTAAAGGCTCCTTTTGGAGCCTTTTTTTTGGAGATTTTCAACGTGAAAAAATTATTATTCGCAATTCCTTTAGTTGTTCCTTTCTATTCTCACTCCGCTGAAACTGTTGAAAGTTGTTTAGCAAAATCCCATACAGAAAATTCATTTACTAACGTCTGGAAAGACGACAAAACTTTAGATCGTTACGCTAACTATGAGGGCTGTCTGTGGAATGCTACAGGCGTTGTAGTTTGTACTGGTGACGAAACTCAGTGTTACGGTACATGGGTTCCTATTGGGCTTGCTATCCCTGAAAATGAGGGTGGTGGCTCTGAGGGTGGCGGTTCTGAGGGTGGCGGTTCTGAGGGTGGCGGTACTAAACCTCCTGAGTACGGTGATACACCTATTCCGGGCTATACTTATATCAACCCTCTCGACGGCACTTATCCGCCTGGTACTGAGCAAAACCCCGCTAATCCTAATCCTTCTCTTGAGGAGTCTCAGCCTCTTAATACTTTCATGTTTCAGAATAATAGGTTCCGAAATAGGCAGGGGGCATTAACTGTTTATACGGGCACTGTTACTCAAGGCACTGACCCCGTTAAAACTTATTACCAGTACACTCCTGTATCATCAAAAGCCATGTATGACGCTTACTGGAACGGTAAATTCAGAGACTGCGCTTTCCATTCTGGCTTTAATGAGGATTTATTTGTTTGTGAATATCAAGGCCAATCGTCTGACCTGCCTCAACCTCCTGTCAATGCTGGCGGCGGCTCTGGTGGTGGTTCTGGTGGCGGCTCTGAGGGTGGTGGCTCTGAGGGTGGCGGTTCTGAGGGTGGCGGCTCTGAGGGAGGCGGTTCCGGTGGTGGCTCTGGTTCCGGTGATTTTGATTATGAAAAGATGGCAAACGCTAATAAGGGGGCTATGACCGAAAATGCCGATGAAAACGCGCTACAGTCTGACGCTAAAGGCAAACTTGATTCTGTCGCTACTGATTACGGTGCTGCTATCGATGGTTTCATTGGTGACGTTTCCGGCCTTGCTAATGGTAATGGTGCTACTGGTGATTTTGCTGGCTCTAATTCCCAAATGGCTCAAGTCGGTGACGGTGATAATTCACCTTTAATGAATAATTTCCGTCAATATTTACCTTCCCTCCCTCAATCGGTTGAATGTCGCCCTTTTGTCTTTGGCGCTGGTAAACCATATGAATTTTCTATTGATTGTGACAAAATAAACTTATTCCGTGGTGTCTTTGCGTTTCTTTTATATGTTGCCACCTTTATGTATGTATTTTCTACGTTTGCTAACATACTGCGTAATAAGGAGTCTTAATCATGCCAGTTCTTTTGGGTATTCCGTTATTATTGCGTTTCCTCGGTTTCCTTCTGGTAACTTTGTTCGGCTATCTGCTTACTTTTCTTAAAAAGGGCTTCGGTAAGATAGCTATTGCTATTTCATTGTTTCTTGCTCTTATTATTGGGCTTAACTCAATTCTTGTGGGTTATCTCTCTGATATTAGCGCTCAATTACCCTCTGACTTTGTTCAGGGTGTTCAGTTAATTCTCCCGTCTAATGCGCTTCCCTGTTTTTATGTTATTCTCTCTGTAAAGGCTGCTATTTTCATTTTTGACGTTAAACAAAAAATCGTTTCTTATTTGGATTGGGATAAATAATATGGCTGTTTATTTTGTAACTGGCAAATTAGGCTCTGGAAAGACGCTCGTTAGCGTTGGTAAGATTCAGGATAAAATTGTAGCTGGGTGCAAAATAGCAACTAATCTTGATTTAAGGCTTCAAAACCTCCCGCAAGTCGGGAGGTTCGCTAAAACGCCTCGCGTTCTTAGAATACCGGATAAGCCTTCTATATCTGATTTGCTTGCTATTGGGCGCGGTAATGATTCCTACGATGAAAATAAAAACGGCTTGCTTGTTCTCGATGAGTGCGGTACTTGGTTTAATACCCGTTCTTGGAATGATAAGGAAAGACAGCCGATTATTGATTGGTTTCTACATGCTCGTAAATTAGGATGGGATATTATTTTTCTTGTTCAGGACTTATCTATTGTTGATAAACAGGCGCGTTCTGCATTAGCTGAACATGTTGTTTATTGTCGTCGTCTGGACAGAATTACTTTACCTTTTGTCGGTACTTTATATTCTCTTATTACTGGCTCGAAAATGCCTCTGCCTAAATTACATGTTGGCGTTGTTAAATATGGCGATTCTCAATTAAGCCCTACTGTTGAGCGTTGGCTTTATACTGGTAAGAATTTGTATAACGCATATGATACTAAACAGGCTTTTTCTAGTAATTATGATTCCGGTGTTTATTCTTATTTAACGCCTTATTTATCACACGGTCGGTATTTCAAACCATTAAATTTAGGTCAGAAGATGAAATTAACTAAAATATATTTGAAAAAGTTTTCTCGCGTTCTTTGTCTTGCGATTGGATTTGCATCAGCATTTACATATAGTTATATAACCCAACCTAAGCCGGAGGTTAAAAAGGTAGTCTCTCAGACCTATGATTTTGATAAATTCACTATTGACTCTTCTCAGCGTCTTAATCTAAGCTATCGCTATGTTTTCAAGGATTCTAAGGGAAAATTAATTAATAGCGACGATTTACAGAAGCAAGGTTATTCACTCACATATATTGATTTATGTACTGTTTCCATTAAAAAAGGTAATTCAAATGAAATTGTTAAATGTAATTAATTTTGTTTTCTTGATGTTTGTTTCATCATCTTCTTTTGCTCAGGTAATTGAAATGAATAATTCGCCTCTGCGCGATTTTGTAACTTGGTATTCAAAGCAATCAGGCGAATCCGTTATTGTTTCTCCCGATGTAAAAGGTACTGTTACTGTATATTCATCTGACGTTAAACCTGAAAATCTACGCAATTTCTTTATTTCTGTTTTACGTGCAAATAATTTTGATATGGTAGGTTCTAACCCTTCCATTATTCAGAAGTATAATCCAAACAATCAGGATTATATTGATGAATTGCCATCATCTGATAATCAGGAATATGATGATAATTCCGCTCCTTCTGGTGGTTTCTTTGTTCCGCAAAATGATAATGTTACTCAAACTTTTAAAATTAATAACGTTCGGGCAAAGGATTTAATACGAGTTGTCGAATTGTTTGTAAAGTCTAATACTTCTAAATCCTCAAATGTATTATCTATTGACGGCTCTAATCTATTAGTTGTTAGTGCTCCTAAAGATATTTTAGATAACCTTCCTCAATTCCTTTCAACTGTTGATTTGCCAACTGACCAGATATTGATTGAGGGTTTGATATTTGAGGTTCAGCAAGGTGATGCTTTAGATTTTTCATTTGCTGCTGGCTCTCAGCGTGGCACTGTTGCAGGCGGTGTTAATACTGACCGCCTCACCTCTGTTTTATCTTCTGCTGGTGGTTCGTTCGGTATTTTTAATGGCGATGTTTTAGGGCTATCAGTTCGCGCATTAAAGACTAATAGCCATTCAAAAATATTGTCTGTGCCACGTATTCTTACGCTTTCAGGTCAGAAGGGTTCTATCTCTGTTGGCCAGAATGTCCCTTTTATTACTGGTCGTGTGACTGGTGAATCTGCCAATGTAAATAATCCATTTCAGACGATTGAGCGTCAAAATGTAGGTATTTCCATGAGCGTTTTTCCTGTTGCAATGGCTGGCGGTAATATTGTTCTGGATATTACCAGCAAGGCCGATAGTTTGAGTTCTTCTACTCAGGCAAGTGATGTTATTACTAATCAAAGAAGTATTGCTACAACGGTTAATTTGCGTGATGGACAGACTCTTTTACTCGGTGGCCTCACTGATTATAAAAACACTTCTCAGGATTCTGGCGTACCGTTCCTGTCTAAAATCCCTTTAATCGGCCTCCTGTTTAGCTCCCGCTCTGATTCTAACGAGGAAAGCACGTTATACGTGCTCGTCAAAGCAACCATAGTACGCGCCCTGTAGCGGCGCATTAAGCGCGGCGGGTGTGGTGGTTACGCGCAGCGTGACCGCTACACTTGCCAGCGCCCTAGCGCCCGCTCCTTTCGCTTTCTTCCCTTCCTTTCTCGCCACGTTCGCCGGCTTTCCCCGTCAAGCTCTAAATCGGGGGCTCCCTTTAGGGTTCCGATTTAGTGCTTTACGGCACCTCGACCCCAAAAAACTTGATTTGGGTGATGGTTCACGTAGTGGGCCATCGCCCTGATAGACGGTTTTTCGCCCTTTGAC

GTTGGAGTCCACGTTCTTTAATAGTGGACTCTTGTTCCAAACTGGAACAACACTCAACCCTATCTCGGGCTATTCTTTTGATTTATAAGGGATTTTGCCGATTTCGGAACCACCATCAAACAGGATTTTCGCCTGCTGGGGCAAACCAGCGTGGACCGCTTGCTGCAACTCTCTCAGGGCCAGGCGGTGAAGGGCAATCAGCTGTTGCCCGTCTCACTGGTGAAAAGAAAAACCACCCTGGCGCCCAATACGCAAACCGCCTCTCCCCGCGCGTTGGCCGATTCATTAATGCAGCTGGCACGACAGGTTTCCCGACTGGAAAGCGGGCAGTGAGCGCAACGCAATTAATGTGAGTTAGCTCACTCATTAGGCACCCCAGGCTTTACACTTTATGCTTCCGGCTCGTATGTTGTGTGGAATTGTGAGCGGATAACAATTTCACACAGGAAACAGCTATGACCATGATTACGAATTCGAGCTCGGTACCCGGGGATCCTCTAGAGTCGACCTGCAGGCATGCAAGCTTGGCACTGGCCGTCGTTTTACAACGTCGTGACTGG

GAAAACCCTGGCGTTACCCAACTTAATCGCCTTGCAGCACATCCCCCTTTCGCCAGCTGGCGTAATAGCGAAGAGGCCCGCACCGATCGCCCTTCCCAACAGTTGCGCAGCCTGAATGGCGAATGGCGCTTTGCCTGGTTTCCGGCACCAGAAGCGGTGCCGGAAAGCTGGCTGGAGTGCGATCTTCCTGAGGCCGATACTGTCGTCGTCCCCTCAAACTGGCAGATGCACGGTTACGATGCGCCCATCTACACCAACGTGACCTATCCCATTACGGTCAATCCGCCGTTTGTTCCCACGGAGAATCCGACGGGTTGTTACTCGCTCACATTTAATGTTGATGAAAGCTGGCTACAGGAAGGCCAGACGCGAATTATTTTTGATGGCGTTCCTATTGGTTAAAAAATGAGCTGATTTAACAAAAATTTAATGCGAATTTTAACAAAATATTAACGTTTACAATTTAAATATTTGCTTATACAATCTTCCTGTTTTTGGGGCTTTTCTGATTATCAACCGGGGTACATATGATTGACATGCTAGTTTTACGATTACCGTTCATCGATTCTCTTGTTTGCTCCAGACTCTCAGGCAATGACCTGATAGCCTTTGTAGATCTCTCAAAAATAGCTACCCTCTCCGGCATTAATTTATCAGCTAGAACGGTTGAATATCATATTGATGGTGATTTGACTGTCTCCGGCCTTTCTCACCCTTTTGAATCTTTACCTACACATTACTCAGGCATTGCATTTAAAATATATGAGGGTTCTAAAAATTTTTATCCTTGCGTTGAAATAAAGGCTTCTCCCGCAAAAGTATTACAGGGTCATAATGTTTTTGGTACAACCGATTTAGCTTTATGCTCTGAGGCTTTATTGCTTAATTTTGCTAATTCTTTGCCTTGCCTGTATGATTTATTGGATGTT

S2. Computer-aided design (CAD) of four DNA-origami nanostructures (DONs)

After pre-designing the draft (**Figure S1**), for details of the design blueprint), the blueprint was drawn in the software by selecting the honeycomb lattice mode through the caDNAno software (2.4.0, Wyss Institute, Harvard University)[1, 2]. Scaffolds with different cross-sectional circles were joined into a complete line with a total length of 7249 nucleotides by creating crossover points at predetermined folding points (the appropriate crossover position was selected according to the helical geometry of the DNA). The backbone was added to the scaffold strand. It was cut into fragments of approximately 20 to 60 bases in length using a break tool (**Figure S2.1~4**).

MD simulations of the designed caDNAno drawings were subsequently performed by TacoxDNA (Webserver: http://tacoxdna.sissa.it/cadnano_oxDNA, Institute for Computational Molecular Science, Temple University) and oxDNA (Webserver: https://oxdna.org/static/oxdna-viewer/index.html, oxDNA2.0, University of Oxford)[3]. The simulation steps were as follows: Conversion to oxDNA format: Use TacoxDNA to convert the design file into an initial configuration in the oxDNA format (.top and .oxdna file). This could be done through the web interface. Relaxation of initial geometry: Run a minimization algorithm to remove particle overlaps and overstretched bonds. This could be done by setting *sim_type = min* in the input file and running the command *oxDNA input_min*. This was followed by a molecular dynamics simulation using a modified backbone potential to allow for larger scale motions. This could be run on a GPU and is specified by *sim_type = relax* in the input file, followed by the command *oxDNA input_relax*. Visualization: Use oxView to visualize configurations and manipulate trajectories.


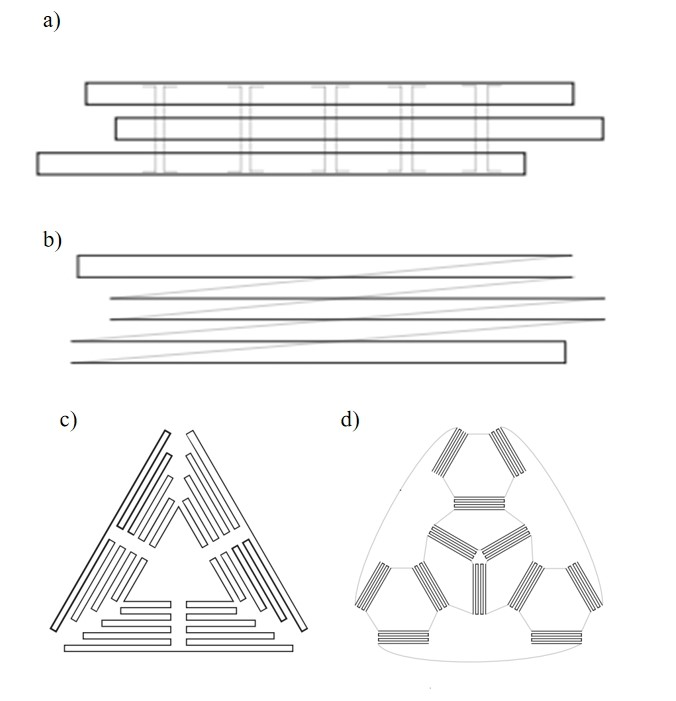


**Figure S.1** Outline of the desired DNA-origami shapes using the caDNAno. General route of scaffolded DNA-origami into a) rod, b) ring, c) triangle and d) octahedron. The continuous scaffolded linear ssDNA M13mp18 (7249 n.t.) was routed throughout the entire desired shape by creating crossovers. Desired DNA-origami shapes were obtained by adding ssDNA staples. The dark line represented the actual DNA scaffold route.


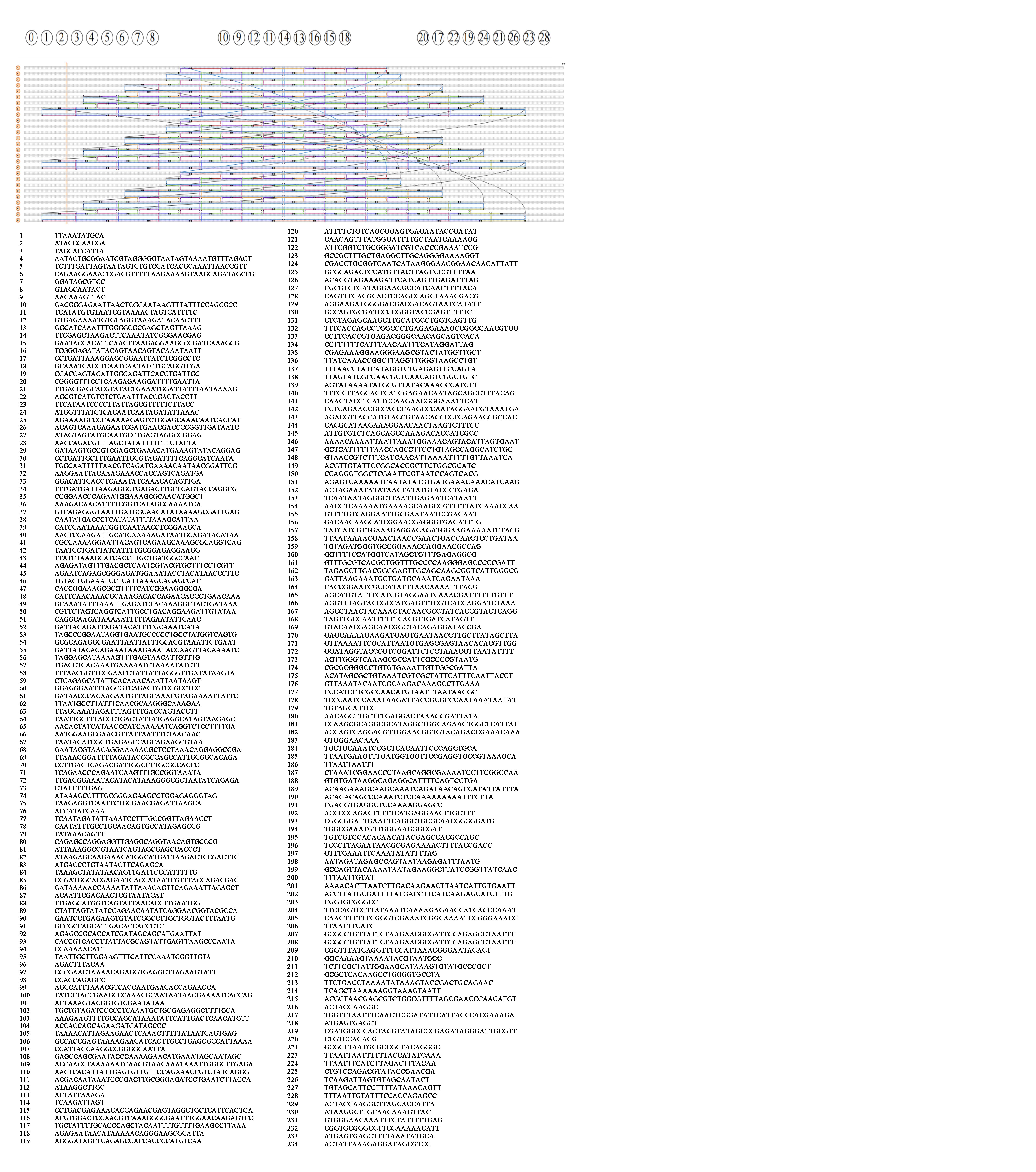


**Figure S2.1** Illustration of triangle DONs caDNAno diagram. Blue line was scaffold strand, and other color line were staples strands;


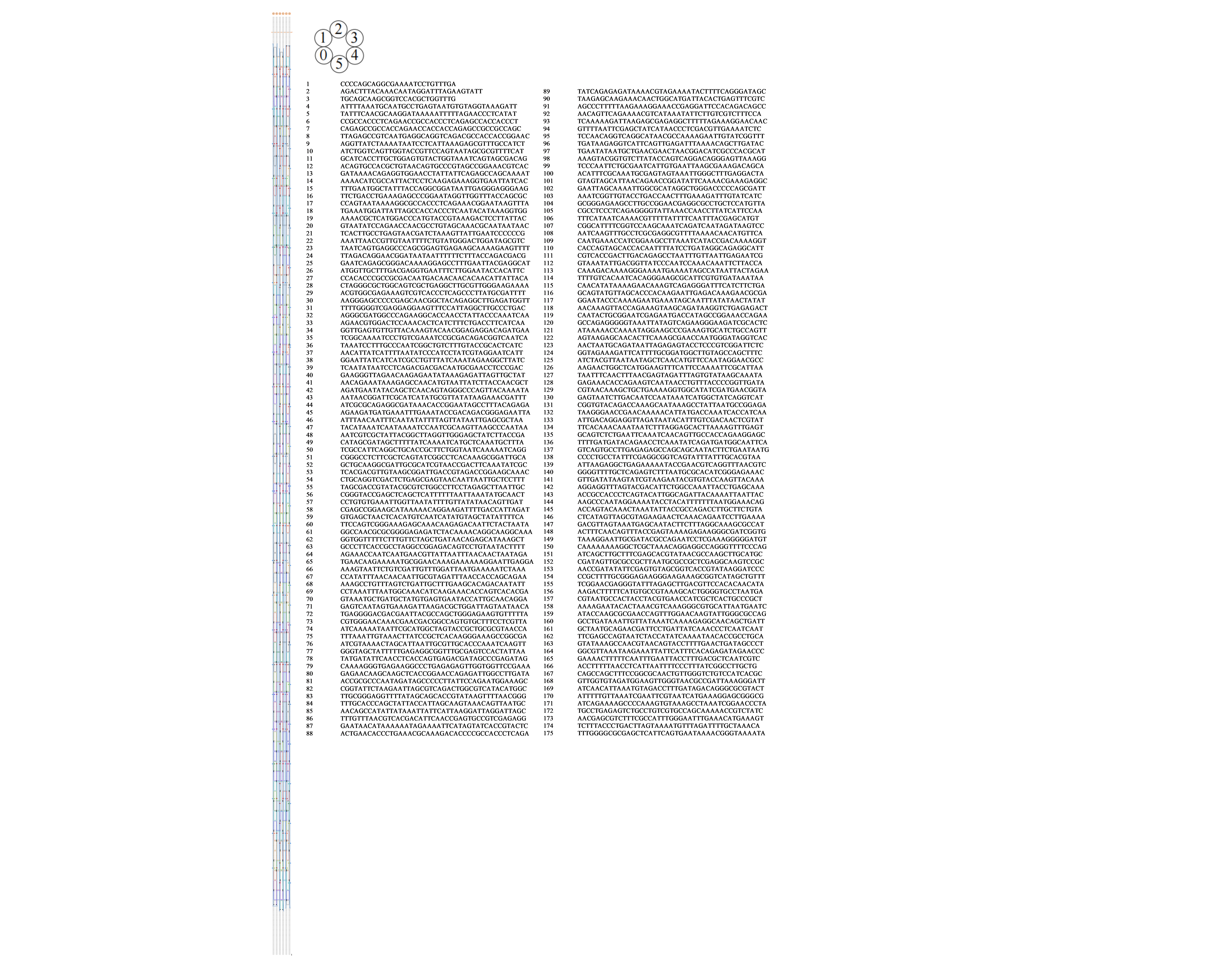


**Figure S2.2** Illustration of rod DONs caDNAno diagram. Blue line was scaffold strand, and other color line were staples strands.


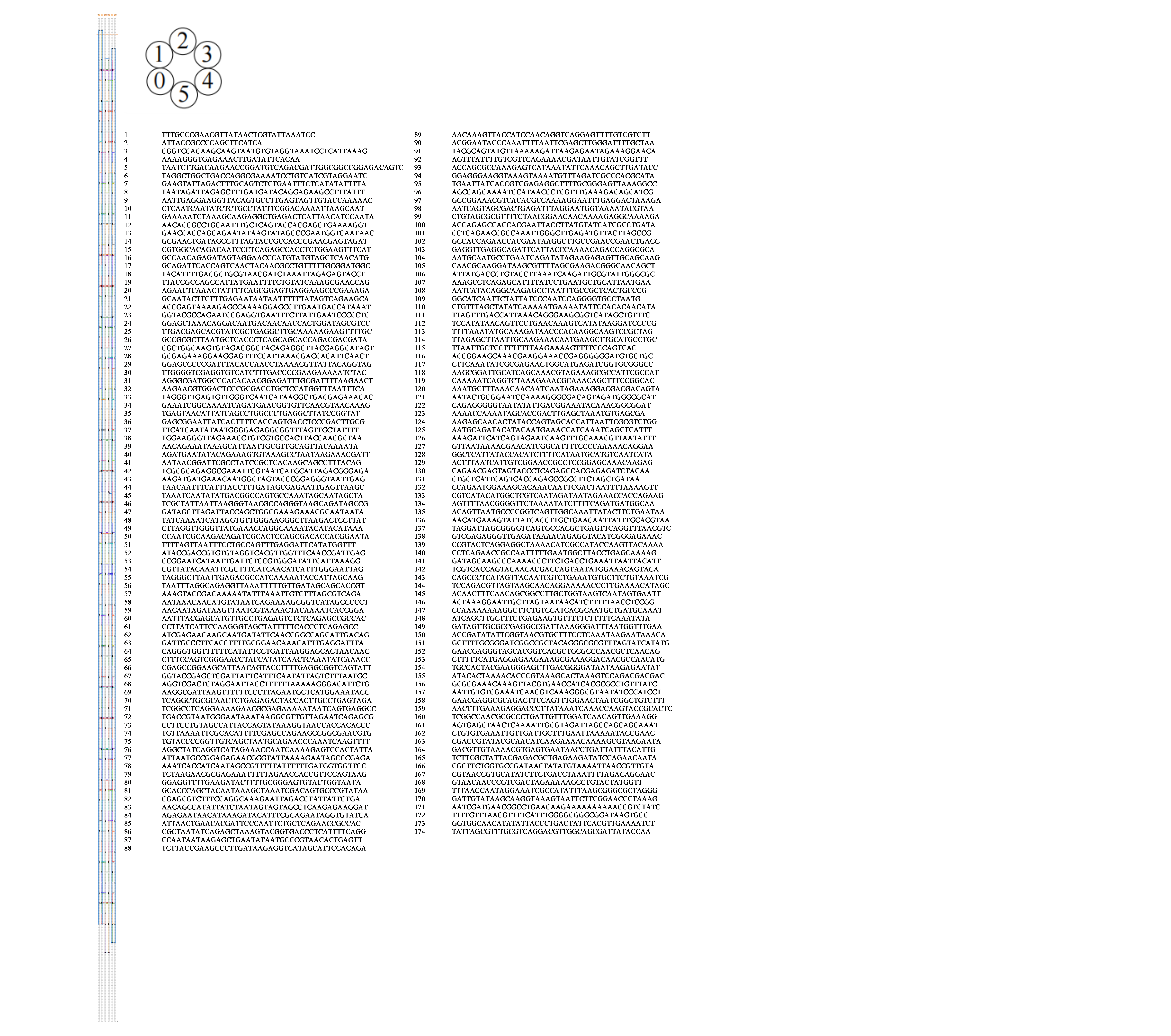


**Figure S2.3** Illustration of ring DONs caDNAno diagram. Blue line was scaffold strand, and other color line were staples strands.


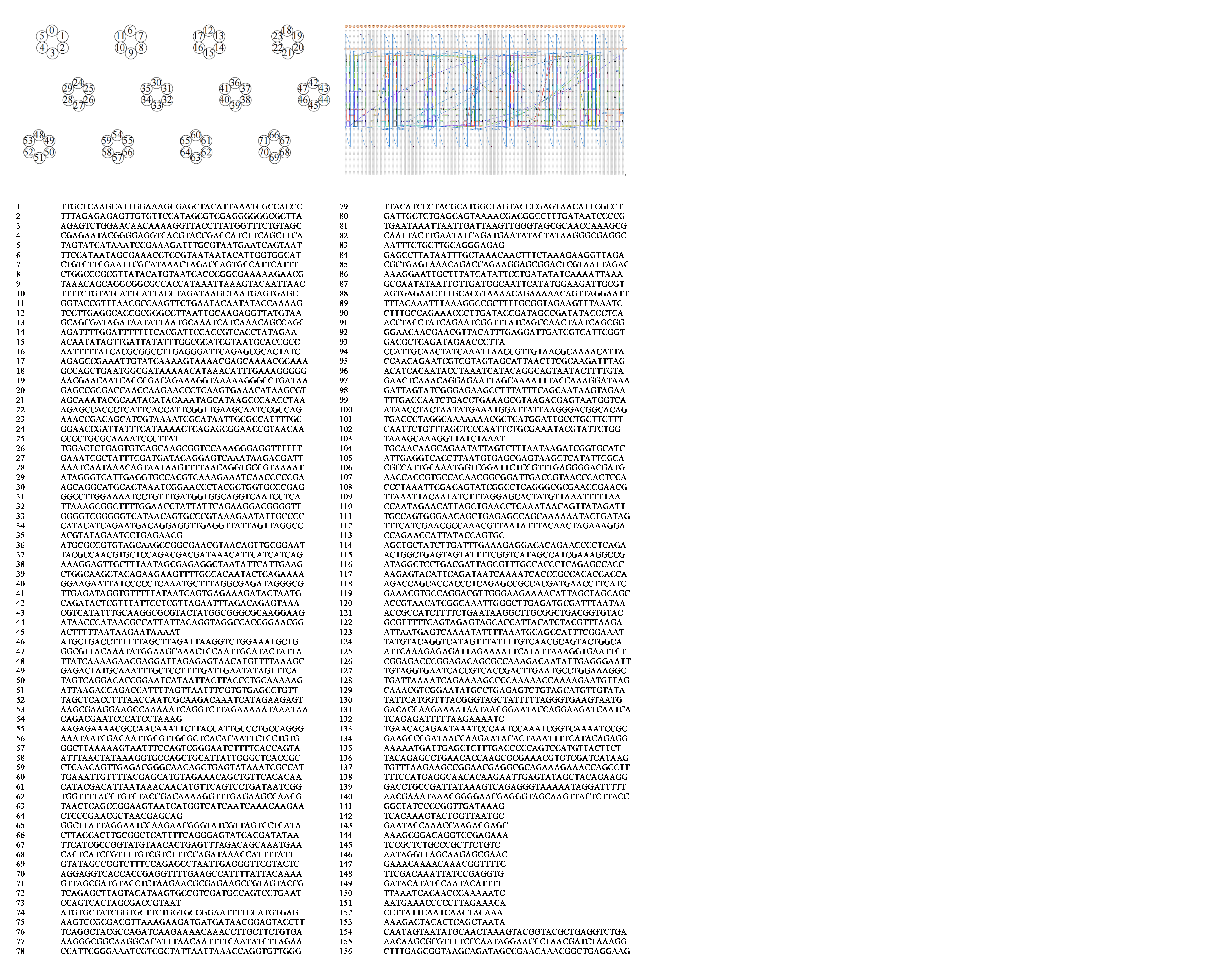


**Figure S2.4** Illustration of octahedron DONs caDNAno diagram. Blue line was scaffold strand, and other color line were staples strands.

S3. DNA Folding reaction recipe for four DONs

Table S3.1 DNA Folding reaction recipe for triangle DONs

| Component | Initial concentration | Volume | Final concentration |
| --- | --- | --- | --- |
| Scaffold (M13mp18) | 100 nM | 1.6 μL | 1.6 nM |
| Staples | 0.43 μM | 37.3 μL | 160 nM |
| MgCl_2_ | 125 mM | 10 μL | 12.5 mM |
| Ultrapure water | / | 41.1 μL | / |
| 10 🞨 TAE | / | 10 μL | / |

Table S3.2 DNA Folding reaction recipe for rod DONs

| Component | Initial concentration | Volume | Final concentration |
| --- | --- | --- | --- |
| Scaffold (M13mp18) | 100 nM | 1.6 μL | 1.6 nM |
| Staples | 0.57 μM | 28 μL | 160 nM |
| MgCl_2_ | 125 mM | 10 μL | 12.5 mM |
| Ultrapure water | / | 50.4 μL | / |
| 10 🞨 TAE | / | 10 μL | / |

Table S3.3 DNA Folding reaction recipe for ring DONs

| Component | Initial concentration | Volume | Final concentration |
| --- | --- | --- | --- |
| Scaffold (M13mp18) | 100 nM | 1.6 μL | 1.6 nM |
| Staples | 0.57 μM | 28 μL | 160 nM |
| MgCl_2_ | 125 mM | 10 μL | 12.5 mM |
| Ultrapure water | / | 50.4 μL | / |
| 10 🞨 TAE | / | 10 μL | / |

Table S3.4 DNA Folding reaction recipe for octahedron DONs

| Component | Initial concentration | Volume | Final concentration |
| --- | --- | --- | --- |
| Scaffold (M13mp18) | 100 nM | 1.6 μL | 1.6 nM |
| Staples | 0.64 μM | 25 μL | 160 nM |
| MgCl_2_ | 125 mM | 10 μL | 12.5 mM |
| Ultrapure water | / | 53.4 μL | / |
| 10 🞨 TAE | / | 10 μL | / |

S4. Yield% calculation for each DONs

The theoretical value was calculated based on the ssDNA scaffold concentration (1.6 nM). Each n.t. was assumed to have an average molecular weight of 330 g/mol (**Suppl. Mater. Excel S2**). Nanodrop was used to measure experimental concentrations of DNA-origami samples. One μL of purified individual DNA-origami nanostructure was dropped onto the sample detection platform, the droplet was forced into the center detection chamber of the platform by lowering the cantilever to form a liquid column. The double-stranded DNA detection module was selected, and the system default parameters were chosen as follow: a baseline corrected wavelength of 340 nm, a default double-stranded extinction coefficient of 50 ng-cm/μL, and the A260/A230 and A260/A280 ratios were used to validate the DNA purity. The final measurement of the sample concentration was recorded in ng/μL (**Table S4**).

Table S.4 Yield% of DNA-origami nanostructures measured by Nanodrop

| DNA nanostructure | Measured concentration (ng/μL) | Estimated theoretical value (ng/μL) | Yield^a^ (%) |
| --- | --- | --- | --- |
| Triangle | 5.0 ± 0.17 | 7.78 | 56 |
| Ring | 4.2 ± 0.21 | 7.66 | 55 |
| Rod | 4.5 ± 0.12 | 7.68 | 59 |
| Octahedron | 3.9 ± 0.6 | 7.02 | 54 |

a: $Yield=\frac{Measured concentration}{Theoretical value}\times100\%$

S5. Purification efficiency using ultracentrifugation

Ultracentrifugation greatly reduced the excess stapled DNA in the DONs samples. To estimate the purification, we used Image J to quantify the OD values of each band on the agarose gel before and after ultracentrifugation (**Table S5**).

Table S.5 Purification of DONs (n=6)

|  | Before purification | | After purification | |
| --- | --- | --- | --- | --- |
|  | DNA origami ^a^ | staple DNA ^b^ | DNA origami ^a^ | staple DNA ^b^ |
| triangle | 8.04±1.04 | 91.96±1.04 | 82.73±15.04 | 17.27±15.04 |
| rod | 8.36±0.90 | 91.64±0.90 | 83.07±21.18 | 16.93±21.18 |
| ring | 5.99±4.25 | 94.01±4.25 | 81.84±14.17 | 18.16±14.17 |
| octahedron | 5.40±1.27 | 94.60±1.27 | 73.50±32.10 | 26.50±32.10 |

a : $Purity\%=\frac{OD of DNA-Origami Structure}{OD of sum [DNA-origami structure + staple DNA]}\times100\%$

b : $Purity\%=\frac{OD of Staple DNA}{OD of sum [DNA-origami structure + staple DNA]}\times100\%$

S6. A fluorescence calibration of Cy5-labelled DONs

To prepare the standard curve, the staple strand labelled with Cy5 (Sangon Biotech, China) were diluted using a serial dilution method (including concentration points of 430 nM, 215 nM, 107.5 nM, 53.75 nM, 26.9 nM). 10 μL of individual DNA-origami nanostructure was added into each well of a 384-well plate. The multifunctional enzyme-linked immunosorbent assay reader (SPARK, Tecan, Switzerland) was used to detect the fluorescence intensity corresponding to different concentrations of Cy5 (630 nm for excitation, 670 nm for emission). This standard curve was used to calculate the concentration of Cy5 in the prepared Cy5-labelled DONs, thus determining the grafting rate of Cy5 in each DONs. The Cy5-labeled staples numbers in the four DONs are:

triangles:

5(TCTTTGATTAGTAATAGTCTGTCCATCACGCAAATTAACCGTT), 111(ACGACAATAAATCCCGACTTGCGGGAGATCCTGAATCTTACCA), 201(AAAACACTTAATCTTGACAAGAACTTAATCATTGTGAATT);

rod:

20(GTAATATCCAGAACCAACGCCTGTAGCAAACGCAATAATAAC), 71(GTAAATGCTGATGCTATGTGAGTGAATACCATTGCAACAGGA), 130(GAGTAATCTTGACAATCCAATAAATCATGGCTATCAGGTCAT);

ring:

20(AGAACTCAAACTATTTTCAGCGGAGTGAGGAAGCCCGAAAGA), 71(TCAGGCTGCGCAACTCTGAGAGACTACCACTTGCCTGAGTAGA), 130(CAGAACGAGTAGTACCCTCAGAGCCACGAGAGATCTACAA); octahedron: 34(CATACATCAGAATGACAGGAGGTTGAGGTTATTAGTTAGGCC), 85(CGCTGAGTAAACAGACCAGAAGGAGCGGACTCGTAATTAGAC), 135(AAAAATGATTGAGCTCTTTGACCCCCAGTCCATGTTACTTCT) .


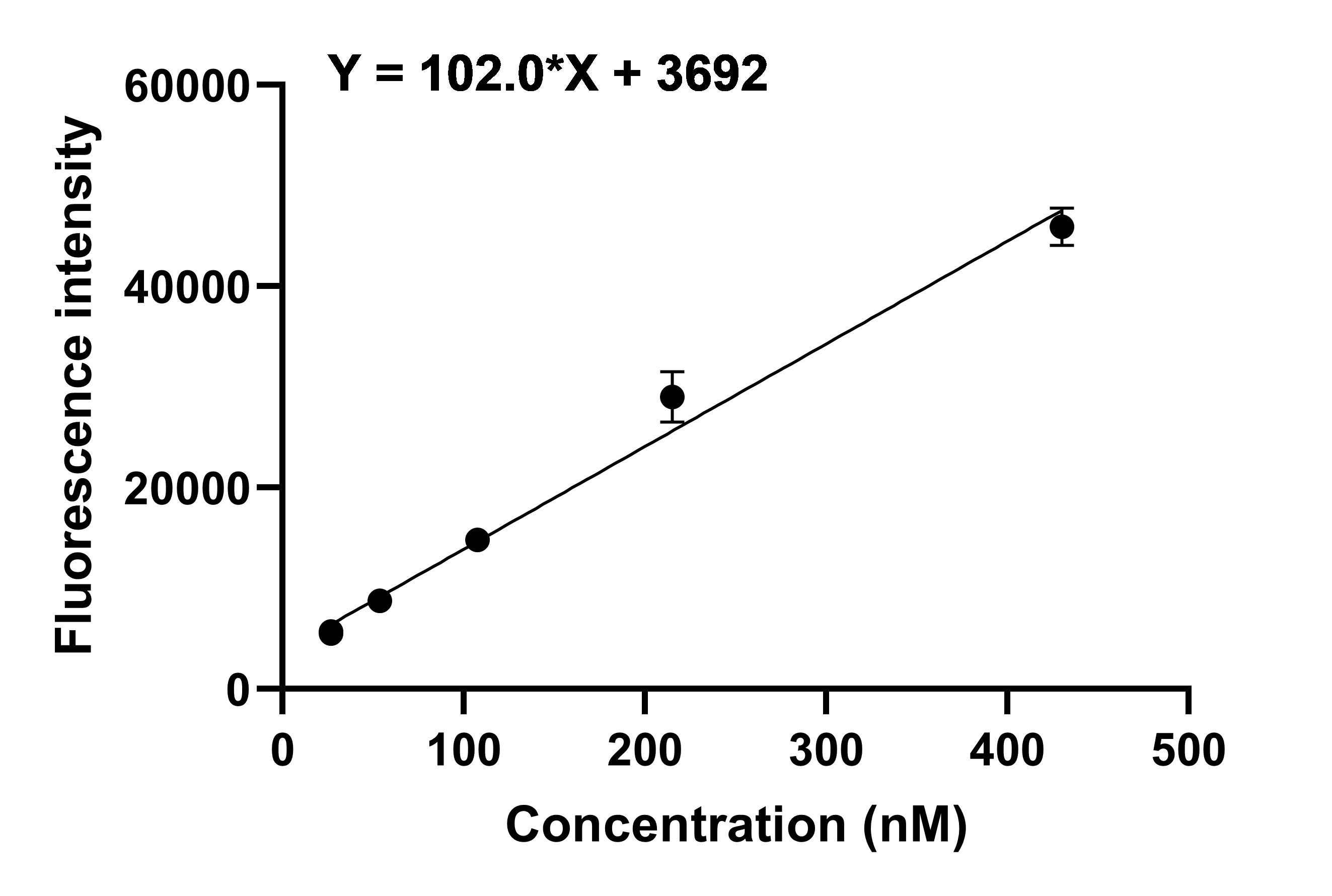


**Figure S.6** A standard curve of fluorescence intensity vs. Cy5 labeled staple over a concentration range of 26.9nM to 430nM (n=6).

S7. Concentration of Cy5-labelled DONs

The concentration of each Cy5-labelled DONs was measured by NanoDrop^TM^One^C^, and the fluorescence intensity of Cy5-labelled DNA nanostructures was detected by the multifunctional enzyme-linked immunosorbent assay reader. The grafting rate of the structure can be calculated by dividing the concentration of Cy5 in Cy5-labelled DNA nanostructure by the concentration of the DNA structure. In order to balance the difference in the grafting rates of the four structures, a correction factor was used to calibrate the fluorescence intensity of the four structure. The correction factor of each Cy5-labelled DNA nanostructure was calculated according to the following equation:

$Correction factor=\frac{F\mathrm{luore} scence intensity of DNA structure}{Fluorescence intensity of Triangle}\times\frac{Molar concentration of DNA structure}{Molar concentration of Triangle}$

Table S.7 Correction factor of Cy5-labelled DNA nanostructures

| Origami nanostructure | molar concentration (nM) | Fluorescence intensity | Correction factor |
| --- | --- | --- | --- |
| Triangle | 0.96 ± 0.08 | 37790 ± 2977 | 1.0 |
| Rod | 0.92 ± 0.03 | 49362 ± 944 | 1.3 |
| Ring | 0.94 ± 0.04 | 38168 ± 1085 | 1.0 |
| Octahedron | 0.92 ± 0.03 | 41516 ± 994 | 1.1 |

S8. Flow cytometry determination of cellular uptake of Cy5-labelled DONs


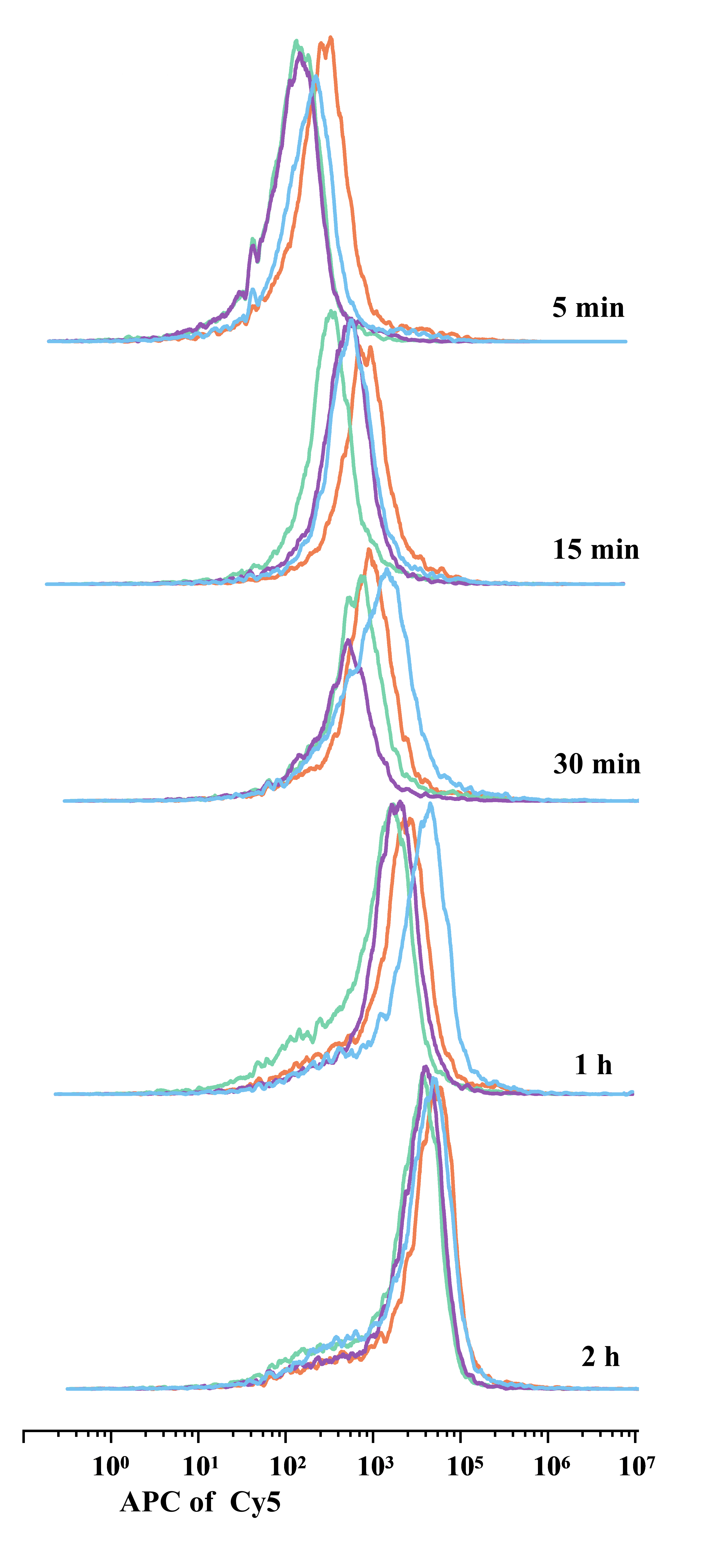


**Figure S.8** Histogram-waterfall plot of cellular uptake of DNA nanostructures in human lens epithelial cells HLE-B3. Flow cytometry analysis of cellular uptake of four DNA nanostructures at pre-determined incubation time points (5, 15, 30, 60, and 120 min) in HLE-B3 cells. The APC channel in the flow cytometer was used to detect Cy5 fluorescence.

S9. PCC analysis of co-localization of Cy5-labelled DONd and cellular organelles in confocal images

To quantitatively determine whether DNA nanostructures were co-localized at nucleus and mitochondria and their degree of co-localization, the Pearson’s correlation coefficient (PCC) was chosen to describe the fluorescence co-occurrence of Cy5 with mito-tracker and DAPI, respectively. In brief, we used coloc 2 in Image J (https://imagej.net/ij, version 1.51j8, USA). selected 67, 68, 62 and 117 cells for four DNA nanostructure by using ROI manager. Differential biodistribution of four origami shapes at mitochondria and nucleus were shown in Figure S9. One-way ANOVA (GraphPad, unpaired, Gaussian distribution) was performed to compare the difference of means of four DNA nanostructures.

P value between 1 and -1 describes the degree of co-localization of the dye fluorescence of mitochondria or nucleus with each Cy5 labelled DNA nanostructure. 1 denotes the perfect correlation (where there is A, there must be B); -1 denotes the complete exclusion (where there is A, there must be no B), and 0 denotes a stochastic relationship (A and B are randomly distributed and unrelated) [4-7].


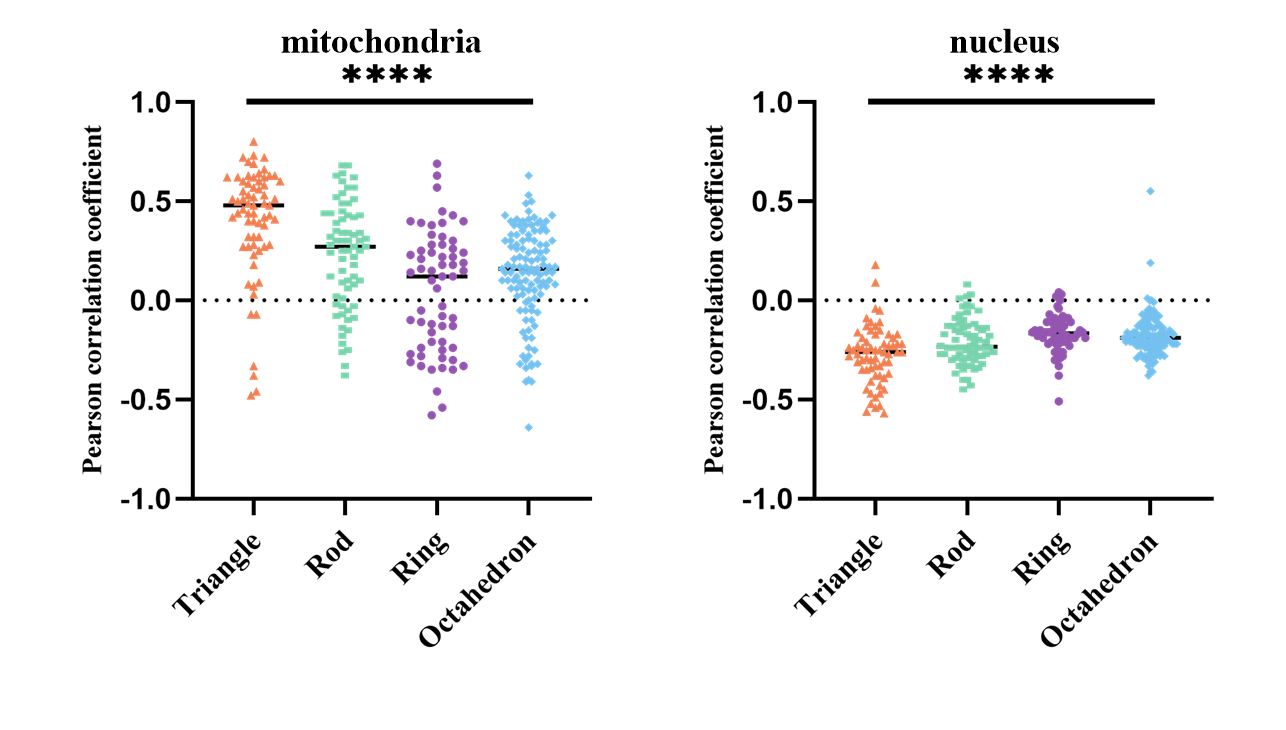


**Figure S.9** PCC distribution of DONs in HLEB-3 cells. The dash line was the mean of PCC. *P<0.05; **P<0.01; ***P<0.001; ****P<0.0001. One-way ANOVA was used to compare means between groups.

S10. Statistical comparison of MCC of mitochondria and nuclei for triangles with other DONs

The data was analyzed based on Figure 7b) (manuscript). Co-localization data of all DONs with mitochondria and nuclei were obtained by MCC analysis, including M1-mito, M2-mito, M1-nuclei and M2-nuclei. Subsequently MMC data (M1 and M2) for the triangles were compared with other DONs using T-test. The results were shown in Table S.10. For mitochondria co-localization, there was significant differences between the triangles and the remaining three structures, while in nucleus, significant difference existed only with the octahedron.

Table S.10 Comparison of MCC for triangles with ones of three other DONs

| T Test^a^ | M1-mito | M2-mito | M1-nuclei | M2-nuclei |
| --- | --- | --- | --- | --- |
| T-Rod | *** | * | / | / |
| T-Ring | **** | **** | ** | / |
| T-Octahedron | **** | ** | **** | **** |

^a: *P<0.05; **P<0.01; ***P<0.001; ****P<0.0001.^

Reference

1. Suma A, Poppleton E, Matthies M, Sulc P, Romano F, Louis AA, et al. TacoxDNA: A user-friendly web server for simulations of complex DNA structures, from single strands to origami. J Comput Chem. 2019;40(29):2586-95. doi: 10.1002/jcc.26029.

2. Douglas SM, Marblestone AH, Teerapittayanon S, Vazquez A, Church GM, Shih WM. Rapid prototyping of 3D DNA-origami shapes with caDNAno. Nucleic Acids Res. 2009;37(15):5001-6. doi: 10.1093/nar/gkp436.

3. Poppleton E, Romero R, Mallya A, Rovigatti L, Šulc P. OxDNA.org: a public webserver for coarse-grained simulations of DNA and RNA nanostructures. Nucleic Acids Res. 2021;49(W1):W491-w8. doi: 10.1093/nar/gkab324.

4. Manders EMM, Verbeek FJ, Aten JA. Measurement of co-localization of objects in dual-colour confocal images. J Microsc. 1993;169(3):375-82. doi: 10.1111/j.1365-2818.1993.tb03313.x.

5. Comeau JWD, Costantino S, Wiseman PW. A Guide to Accurate Fluorescence Microscopy Colocalization Measurements. Biophysical journal. 2006;91(12):4611-22. doi: <https://doi.org/10.1529/biophysj.106.089441>.

6. Dunn KW, Kamocka MM, McDonald JH. A practical guide to evaluating colocalization in biological microscopy. American journal of physiology Cell physiology. 2011;300(4):C723-42. doi: 10.1152/ajpcell.00462.2010.

7. French AP, Mills S, Swarup R, Bennett MJ, Pridmore TP. Colocalization of fluorescent markers in confocal microscope images of plant cells. Nature Protocols. 2008;3(4):619-28. doi: 10.1038/nprot.2008.31.
